# Use and valuation of native and introduced medicinal plant species in Campo Hermoso and Zetaquira, Boyacá, Colombia

**DOI:** 10.1186/1746-4269-9-23

**Published:** 2013-04-11

**Authors:** Ana Lucía Cadena-González, Marten Sørensen, Ida Theilade

**Affiliations:** 1 Department of Agriculture and Ecology, University of Copenhagen, Rolighedsvej 21, 1958 Frederiksberg C, Denmark; 2 Department of Plant and Environmental Sciences, University of Copenhagen, Rolighedsvej 21, 1958 Frederiksberg C, Denmark; 3 Department of Food and Resource Economics, Faculty of Science, University of Copenhagen, Rolighedsvej 23, 1958 Frederiksberg C, Denmark

**Keywords:** Acculturation, Ethnobotany, Trained healers, Amateur healers, Introduced medicinal plants, Traditional knowledge

## Abstract

**Background:**

Medicinal plant species contribute significantly to folk medicine in Colombia. However, few local studies have investigated whether species used are introduced or native and whether there is a difference in importance of native and introduced medicinal plant species. The aim of the present study was to describe the use of medicinal plants within two municipalities, Campo Hermoso and Zetaquira, both in the department of Boyacá, Colombia and to assess the importance of native and introduced plants to healers, amateur healers and local people. As local healers including amateur healers have no history of introduced species our working hypotheses (H_1-2_) were that H_1_: native and introduced medicinal plant species are of equal importance and H_2_: healers and amateur healers do not differentiate in their preferences between native and introduced medicinal plant species.

**Methods:**

Ten villages were included in the study. A combination of quantitative and qualitative methods was used including questionnaires, semi-structured interviews, in- depth interviews, and open talks. Voucher specimens were collected in home gardens and during field walks. For data analysis, we calculated use value indices and Jaccard index and tested for the above hypothesis using Spearman rank-correlation coefficients and Wilcoxon-Mann–Whitney tests.

**Results:**

Eighty medicinal plant species were described by locals as the most frequently used. Of these, 78 species were taxonomically identified, distributed within 41 families and 74 genera, which included 35 native species and 43 introduced. The highest valued families were: Asteraceae, Lamiaceae, Apiaceae, Rutaceae and Verbenaceae. The species ranked highest according to their Use Values, in both municipalities, were *Mentha suaveolens* Ehrh., *Ambrosia cumanensis* Kunth, and *Verbena littoralis* Kunth. Introduced species were more important than native ones in Zetaquira, while there was no difference in importance in Campo Hermoso. While healers relied most on the uses of native species, amateur healers were inclined to rely on introduced species. Medicinal plant administration in both municipalities follow the usual pattern: Leaves are used most commonly prepared by decoction or infusion and administrated orally.

**Conclusions:**

The high proportion of introduced plant species used in the local traditional medicines is similar to the results of a number of other ethnobotanical studies and emphasise the need for efforts to record and maintain traditional knowledge on native species.

## Background

Medicinal plant species constitute important alternatives to conventional medicine in a large number of developing countries, especially within poor communities that inhabit rural areas and lack access to health services [1,2]. A number of native medicinal plants of both the palaeotropics and neotropics have traditionally had a high value for indigenous communities, not only because of their healing properties, but also due to other uses [3]. An example of such multi-use plants is the neotropical species *Crescentia cujete* L., a source of both timber and traditional medicine. Recently, this species has reached the United States and European pharmaceutical markets demonstrating that traditional medicinal plants used by indigenous communities may be of high economic value [3].

However, knowledge, cultivation and maintenance of these native species within rural communities is decreasing, due to modernization processes, such as acculturation [1-3]. In addition, a tendency to consider all plant resources as native by local people has been directly or indirectly documented in several studies [4-6]; in other words, numerous introduced plant species with healing properties have become popularly considered as ‘native’. Consequently, a number of native medicinal plant species have been replaced by introduced species. Thus, they use the terms ‘native’ and ‘introduced’ indiscriminately so that many introduced species with healing properties have become popularly known as native. For these reasons, the uses of a number of native species have been replaced by introduced species, incorrectly perceived as native.

When faced with the rapid decline in traditional knowledge it is relevant to identify medicinal species and to record their uses within local communities. This is especially important in regions that currently are affected by land-use change and modernisation. Documentation and awareness of ethnobotanical knowledge within these regions may facilitate the maintenance of medicinal plant resources and uses.

Furthermore, ethnobotanical studies may support implementation of strategies that integrate native medicinal plant uses with sustainable management of natural resources. The municipalities of Campo Hermoso and Zetaquira in the department of Boyacá, Colombia, are examples where conversion of forest areas into agriculture land and reforestation with exotic species have destroyed large areas of natural forest including high plateau, upland, and montane forest. Furthermore, many multiple-use plants have been overexploited [7,8]. This situation has caused loss of a number of native species (pers. comm. local key informants 2009). Studies on medicinal plant use have been conducted in different localities within Boyacá [9,10], but none in the regions of Campo Hermoso and Zetaquira. Therefore, the present study was carried out within these municipalities. The aims of this study were to identify the medicinal plant species, to determine their origin (native or introduced to Colombia), to record their medicinal uses (targeted illnesses, ways of administration), and based on personal observations and inspired by previous studies [4-6] to assess the value of native and introduced plant species to members of local communities. The latter objective was evaluated by the null hypotheses (H_1-2_) H_1_: native and introduced medicinal plant species are of equal importance and H_2_: healers and amateur healers do not differentiate in their preferences between native and introduced medicinal plant species.

## Methods

### Study areas

**Figure 1:**
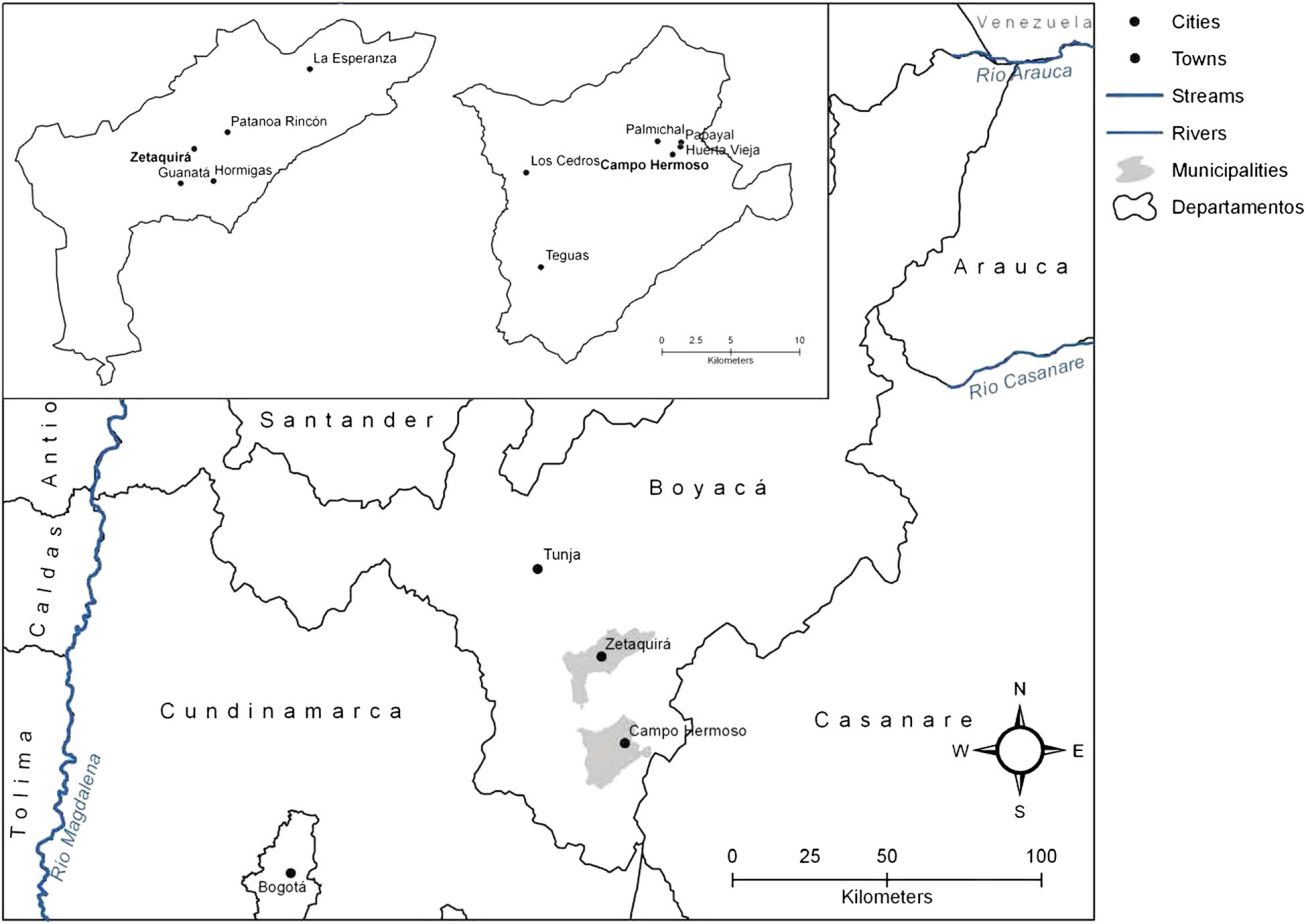
**Main map showing locations of the municipalities of Campo Hermoso and Zetaquira in department of Boyacá.** Inset map: outlines of the municipalities and villages visited in the rural areas. The scale is uniform within the inset map; for true relative positions of the municipalities see main map.

The study took place in the municipalities of Campo Hermoso and Zetaquira in department of Boyacá, Colombia (Figure 1). The municipalities are located on the Eastern slope of the Cordillera Oriental (Eastern Andean range) and their terrain comprises rugged areas with peaks, steep canyons, and valleys. Both study areas belong to the humid subtropical zone [7,8]. The altitude range from 500–2500 m a.s.l. in Campo Hermoso and 1875–3600 m a.s.l. in Zetaquira. Temperatures range between 12–35°C.

Climatic differences and mountainous topography lead to a diversity of natural vegetation, livestock pastures, and crops within the study areas [7,8]. Campo Hermoso is located at 132 km from Tunja the capital of the department and 143 km from Bogotá. Zetaquira is located at 69 km away from Tunja, and at 126 km away from Bogotá.

The economy of the municipalities is based on cattle ranching and agriculture; the latter often by smallholders practicing subsistence economy. The main products in Zetaquira are coffee, beans, sugarcane, maize, plantain, manioc and ‘arracacha’ (*Arracacia xanthorrhiza* Bancr.) [11]. The coffee production is located near the villages at intermediate altitudes. Until 2008, coffee was a main crop in Zetaquira. However, climate change has decreased feasibility of coffee production in recent years (pers. comm.: Domingo Mendoza, responsible of the Federación Nacional de Cafeteros de Colombia, office in Zetaquira). Campo Hermoso is basically a cattle ranching region with large areas of pastures [7]. Besides, maize, beans, manioc and vegetables and, at smaller scale, sugarcane is cultivated for subsistence. There are no conspicuous economic differences between the two municipalities [7,8].

### Data collection and free, prior informed consent (FPIC)

The field work was conducted from October 2008 to January 2009. Data collection included a combination of quantitative and qualitative methods in order to allow for triangulating and cross-checking [12]. Applied and complementary ethnobotanical methods [13] were used to acquire information on how local people related to the medicinal plant resources in the two municipalities. The project was presented to the local authorities and to the communities during initial meetings. Objectives and methods were approved in both municipalities.

#### Selection of villages and identification of informants

Within each municipality representative villages were visited. Ten villages were chosen according to distance to the main urban centre, the presence of a representative flora, and residential areas of the healers and herbalists, who were purposefully included in the sampling. In Zetaquira the villages studied were: La Esperanza, Guanata, Centro Rural and Hormigas; and in Campo Hermoso: Papayal, Huerta Vieja, Palmichal, Los Cedros, Macanalito and Teguas.

Participation of local people was essential to several stages of the research process [12]. Thus, key informants in the municipalities were contacted in the first stage. Their collaboration was crucial for selecting and planning the visits to the villages.

Each municipality was visited three times during the study in order to establish and maintain communication with the local informants, to identify additional informants, and later to apply quantitative and qualitative tools. The informants from the villages were selected using stratified random sampling. Interviewees in the study all belonged to farmers' communities.

#### Application of questionnaires, in-depth interviews and semi-structured interviews

Questionnaires were designed to collect information on the main local medicinal plant uses. The questions were answered by a selected group (n = 9) of old women in Patanoa Rincon in Zetaquira. Interviews were structured in two groups: in depth interviews and semi-structured interviews, which were done with individual informants in order to maintain data independence [13]. In depth interviews were answered by healers^a^ and amateur healers^b^. In-depth interviews were carried out in order to collect information on their knowledge on plant use and their services offered to locals. Semi-structured interviews addressed farmers (n = 41). Their distribution across age and sex is shown in Table 1. Semi-structured interviews helped to collect data on traditional plant knowledge and use. For all interviews, vouchers of local medicinal plant species was used as a stimulus for interviewees to elaborate on known and used plants [14].

**Table 1:** Distribution of informants who answered semi-structured interviews in the municipalities of Campo Hermoso and Zetaquira, including totals, percentage of interviewees native to the municipality, and age classes

			**Age class**		
**Municipality**	**Total**	**% native**	**20 to 40**	**41 to 60**	**>60**
Campo Hermoso	16	81	1	7	8
Zetaquira	25	80	6	11	8

Homegardens, gardens and solares^c^ were visited during the interviews. Plant specimens were collected together with the informants when obtainable. From the list of mentioned plants, those that were not cultivated at home were grouped in a separate list.

Disease was defined by interviewees as a period of disequilibrium in the body due to deterioration of organs and functions. Disequilibrium could also be caused by bacteria and viral infection. The diseases described during the study were grouped into categories as it is shown in Additional file 1.

#### Field walks

A field walk with key informants was conducted in each municipality in order to identify environmental characteristics of medicinal plant species. The field walk in Campo Hermoso took place along a 5 km transect. In Zetaquira, it was conducted along a 4.5 km transect. Both transects traversed hills and sloping terrain, agricultural lands, and pastures for cattle ranching with some spots of secondary forests.

#### Systematics of plant species

Taxonomic identification was mainly conducted by experts at the Colombian National Herbarium (COL) and at the herbarium of Universidad Tecnológica de Tunja and, the Colombian Amazonian Herbarium (COAH) [15]. Collected specimens were deposited at COL, at the non-profit organization Alexander von Humboldt Biological Resources Research Institute Villa de Leyva branch, Colombia (FMB), and at the Herbarium, Botany Group, Department of Plant and Environmental Sciences, Faculty of Science, University of Copenhagen (CP), for future references. Additionally, specimens were deposited with the local authorities of each municipality, for reference and further studies at the local level.

### Data analysis

Different measures of knowledge described by interviewees can be seen in ethnobotanical research. Therefore, it was decided to carefully evaluate the measure of knowledge to be used in the data analysis of the present study.

Plant species mentioned by the informants could be associated with one or several diseases, with one or more uses, or with both diseases and uses. Hence, it was possible to sum up two-way combinations of plant species (with use or disease) or three-way combinations (with use and disease). Since species often were only associated with one or the other, the different combinations might relate to substantially different measures of knowledge. Further, native and introduced species were distinguished, so that it was possible to observe differences of use between the two groups of plant species.

Spearman rank-correlation coefficients [16,17] of the different indices of interviewees' knowledge were calculated (Additional file 2). As the knowledge indices were highly correlated, (correlation coefficient 0.87) it was decided to use plant-disease-use combinations, because they appeared to be the most comprehensive measure.

In order to assess the interviewees’ valuation of the medicinal plant species, use-value indices (UV) [18] were calculated as follows: The plant-disease-use combinations mentioned by the informants were counted. Use value (UV) was calculated by the formula [19]:

(1) $$\mathrm{UV}=\frac{U}{n}$$

where *U* = plant-disease-use combination, and *n* = total number of interviewees in each municipality. Since the informants were interviewed only once, it was decided to interpret each plant-disease-use combination mentioned by each informant as an event.

Furthermore, estimated UVs and actual UVs were calculated for each species following the procedure described above.

In addition, estimated and actual Family Use Values (FUV) were calculated in order to identify the significance of medicinal plant families in the municipalities. For this purpose, Equation 1 was adapted in accordance with the literature [20]:

(2) $$\mathrm{FUV}=\frac{\mathrm{UVs}}{\left(\mathrm{ns}\right)}$$

where *UVs* = use values of the species, and *ns* = total number of species within each family.

Wilcoxon-Mann–Whitney tests [16,17] were used to test for differences between introduced and native medicinal plant species in their estimated and actual UV within the two municipalities.

Finally, similarities of use of introduced and native medicinal plant species between the municipalities were calculated using the Jaccard Index [21,22].

## Results

### Medicinal plant species and plant characteristics

Interviews and questionnaires supplied a list of a total of 80 medicinal plant species used in the treatments of ailments within the municipalities of Campo Hermoso and Zetaquira. Of these, 78 species were taxonomically identified. Results are based on the 78 identified species of which 35 were native species and 43 introduced (Additional file 3), belonging to 74 genera and 41 floristic families. The families with the highest numbers of species reported as medicinal were: Asteraceae (10 species)*,* Lamiaceae (7)*,* Apiaceae*,* Rutaceae and Verbenaceae (each 4), and Malvaceae*,* Solanaceae and Urticaceae (each 3).

Twenty-nine of the identified species and the genus *Cecropia* Loefl. were included in the list of medicinal plants evaluated and accepted in the Colombian pharmacopeia [23]. Thirteen species were reported in the World Health Organization’s (WHO) monographs on selected medicinal plants [24] (Additional file 3).

During interviews and meetings interviewees were able to reflect about the local resources of medicinal plant species, their importance and, additionally, to discuss factors that may have increased or reduced plant diversity, such as logging, over- exploitation and difficulties of cultivation. Some interviewees reported that they found it difficult to treat certain diseases because they failed to find the plant species needed. An example is *Brownea ariza* Benth., a native species, which locals consider extinct in the Campo Hermoso area. This species was used as a haemostat, i.e. to stop bleeding, and as laxative. Another example is *Juglans neotropica* Diels., a native species, used as fungicide and bactericide, which was reported to be endangered in both municipalities (Table 2, Additional file 3). Due to the lack of samples it was not possible to scientifically identify all the species reported as threatened and disappearing by the interviewees.

**Table 2:** Plant species reported as disappeared or endangered within the municipalities of Campo Hermoso and Zetaquira

	**Campo Hermoso**	**Zetaquira**
Disappeared species	*Brownea ariza*	*Fraxinus udhei*
	*Ananas comosus*	*Schizolobium parahybum*
	*Parietaria officinalis*	*Chrysophyllum colombianum*
	*Furcraea* sp.	‘Alma negra’
	*Malva sp.*	
	‘Gualola’	
	‘Mano de León’	
	‘Bejuco de roca’	
Endangered Species	*Furcraeae macrophylla*	*Citrus limon*
	*Artemisia absinthium*	*Saccharum officinarum*
	*Ocimum campechianum*	*Juglans neotropica*
	*Juglans neotropica*	*Rosmarinus officinalis*
	*Ceroxylum quindiuense*	
	*Cedrela* spp.	

According to informants’ reports and the subsequent calculations of average UVs the most popular medicinal species, in the municipality of Campo Hermoso were *Urtica dioica* (0.88)*, Jaccaranda copaia* (0.81) and *Citrus limon* (0.81) while in Zetaquira the most popular species were *Ruta graveolens* (average UV = 1.16)*, Melissa officinalis* (0.8), and *Aloe vera* (0.6). Across both municipalities, the most common and popular medicinal plant species were *Mentha suaveolens*, *Ambrosia cumanensis* Kunth, and *Verbena littoralis* (Additional file 3).

**Figure 2:**
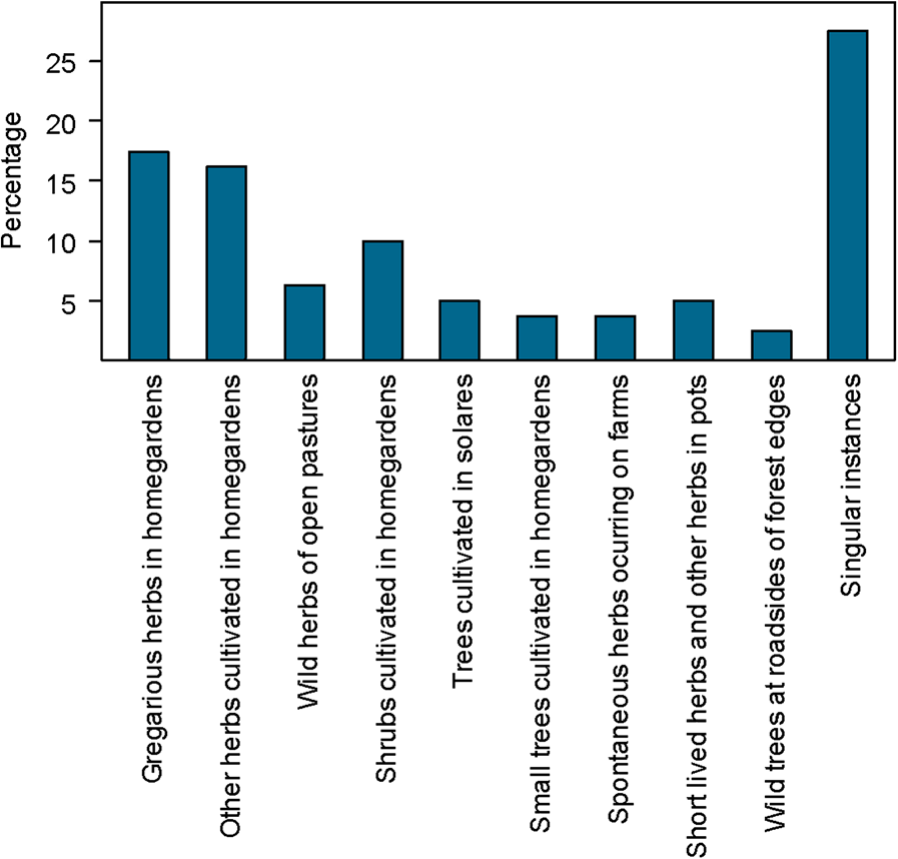
**Life form and habitats of medicinal plant species reported in Campo Hermoso and Zetaquira. ***Gregarious herbs in home gardens* refers to cultivated plants; *Home gardens include also solares; Short-lived herbs and other herbs in pots* are also cultivated; *singular instances* refers to: a wild twining plant along riverbanks, a succulent tall herb on farmland, a wild vine at shady and moist edges of secondary forests, a succulent plant in pots, a tree cultivated in garden.

In relation to life form and habitat, the collected plant species could be assigned to 10 categories shown in the Figure 2 (Additional file 3). Of the native species 40% were found in natural habitats whereas 27% of the introduced species were found as naturalised in the wild.

The naturally occurring and naturalised species were distributed widely within families. For example, Asteraceae (2 herbs), Acanthaceae (1 tree). Aristolochiaceae (1 twiner) and Commelinaceae (2 vines) (Additional file 3).

### Traditional plant use in Campo Hermoso and Zetaquira

**Figure 3:**
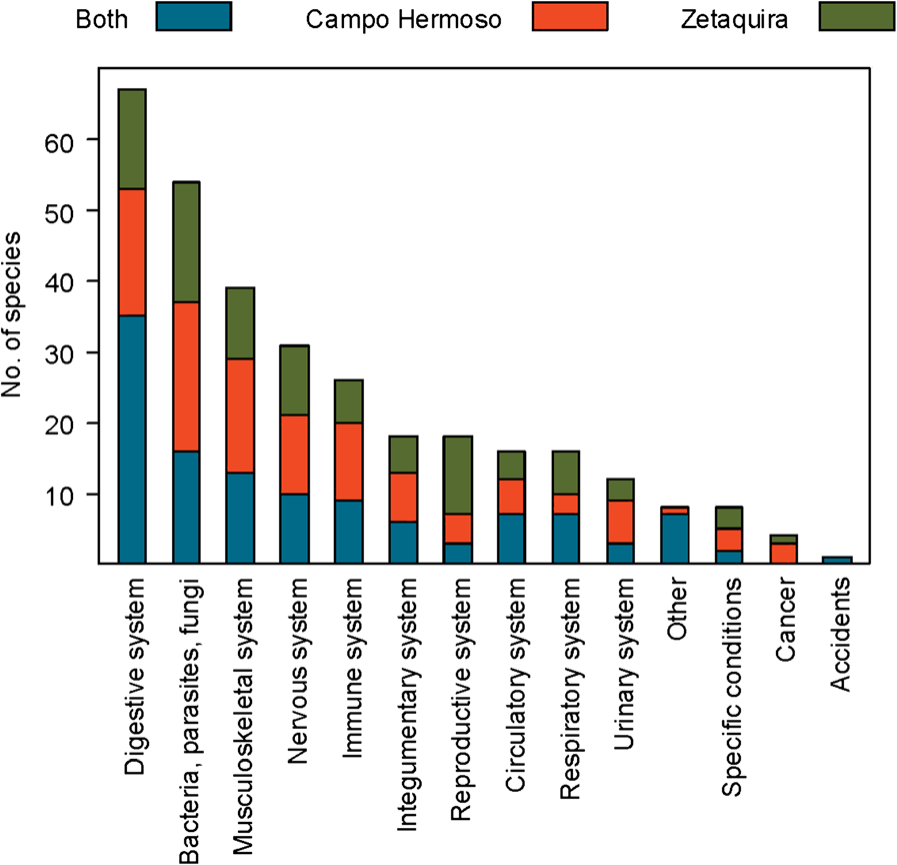
Number of plants used in Campo Hermoso and Zetaquira by categories of diseases.

**Figure 4:**
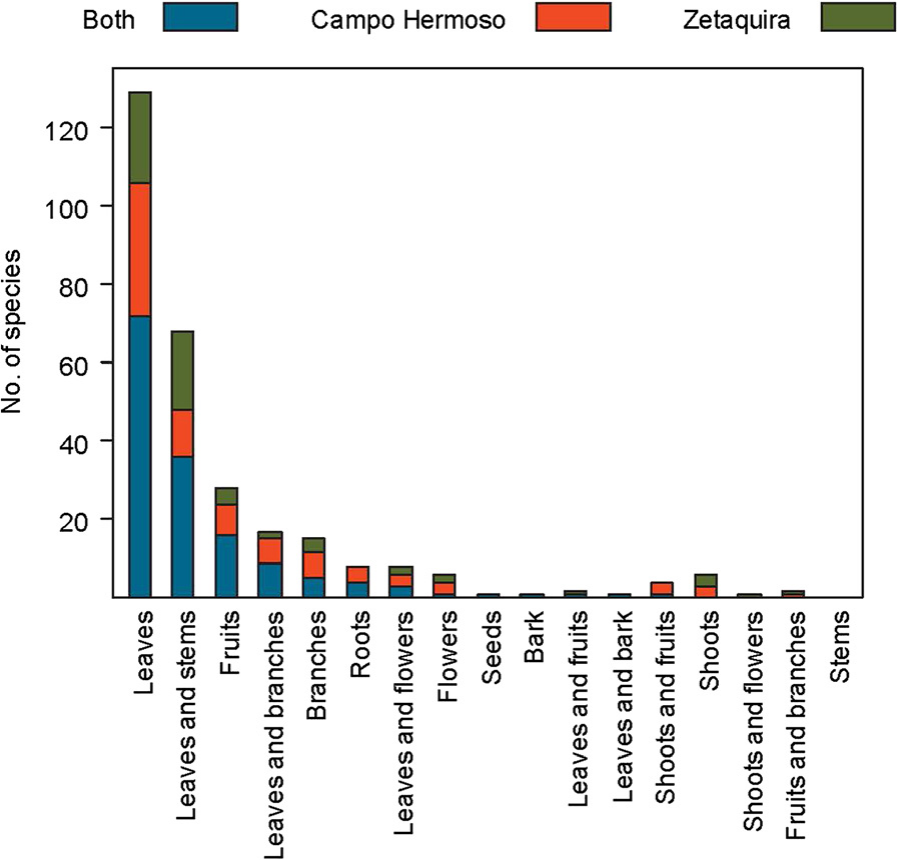
Total numbers of uses of plant parts or combinations of plant parts reported in Campo Hermoso and Zetaquira.

**Figure 5:**
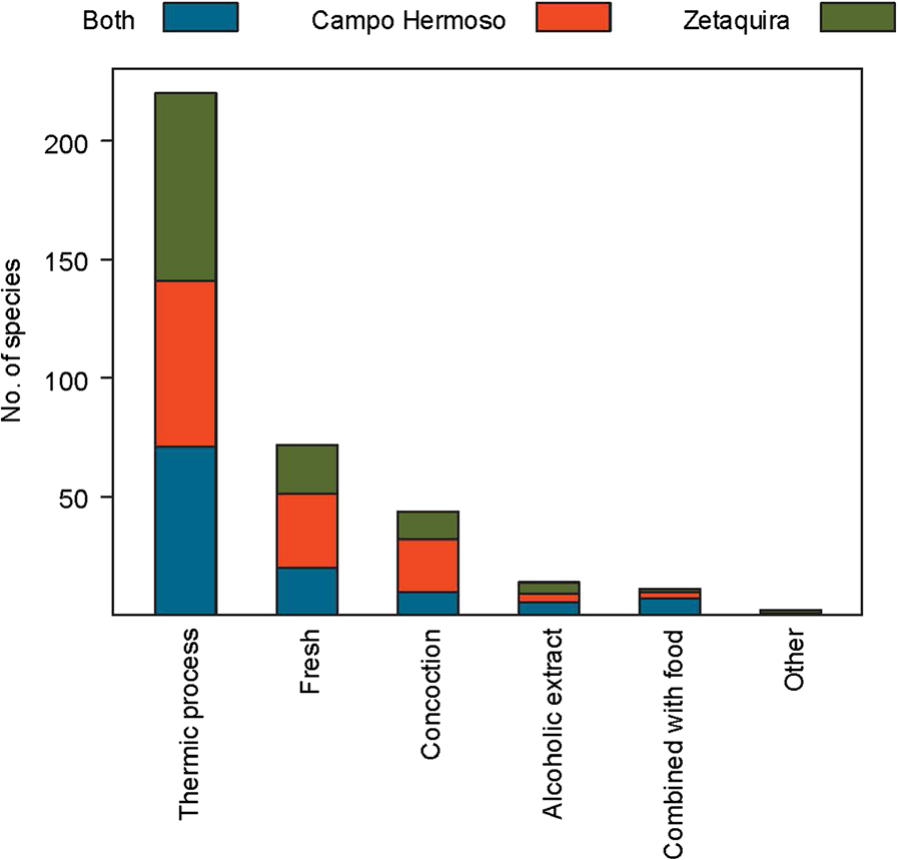
Number of ways of preparation reported for medicinal plants in Campo Hermoso, Zetaquira or in both municipalities.

**Figure 6:**
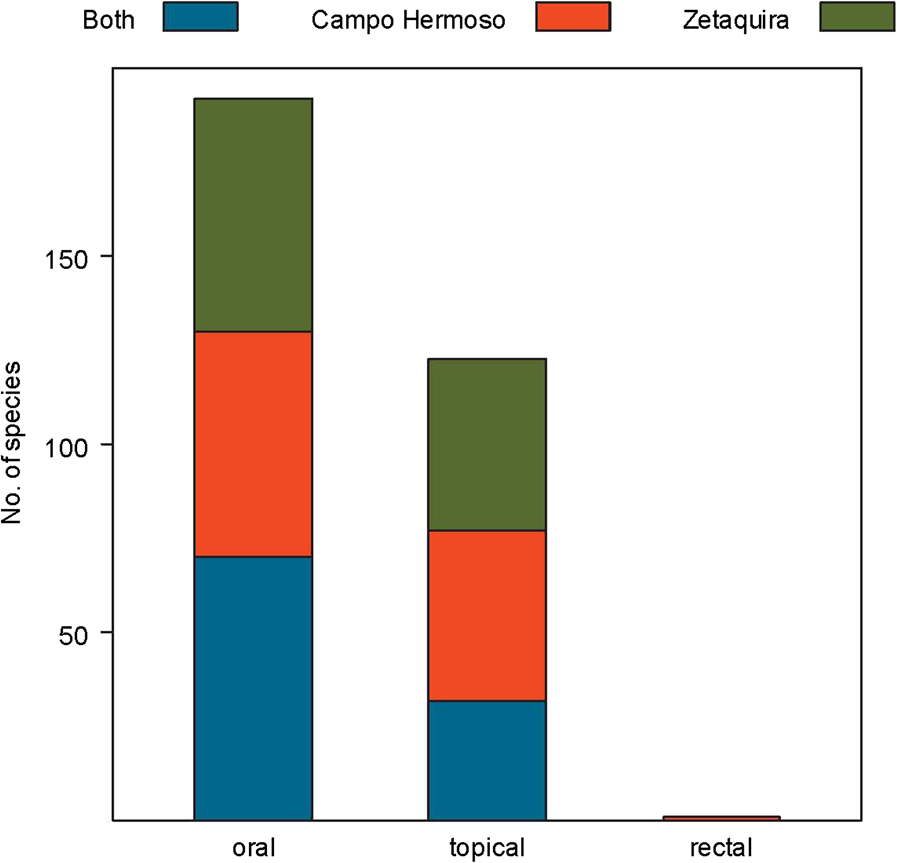
Ways of administration reported in Campo Hermoso, Zetaquira or in both municipalities.

Traditional plant uses in the municipalities of Campo Hermoso and Zetaquira are represented in Figures 3, 4, 5, 6. The total number of medicinal plants uses described by informants was higher in more remote Campo Hermoso than in Zetaquira.

Common diseases, such as colds, but also a small number of difficult or complicated diseases, such as cancer, were reported to be treated. The majority of plants mentioned by the informants were reported to be effective in curing the diseases they were applied to. Additionally, most of the informants mentioned the importance of using folk traditions, e.g. prayers, during treatments in order to ensure the effect of the medicinal plant.

The diseases described by interviewees were grouped in 14 categories (Additional file 1). The five categories of ailments/diseases with the highest numbers of plants reported in both municipalities were associated with: the digestive system, infections, musculoskeletal, the nervous system, and the immune system. The corresponding most popular medicinal plant species are shown in Table 3. Significant dissimilarities between municipalities occurred in treatments of musculoskeletal, immune and urinary systems, and cancer where the total numbers of plants mentioned in more remote Campo Hermoso was almost double the number of Zetaquira. The category with the highest number of medicinal plant species reported from Zetaquira was ‘reproductive system’ with 11 plants. There were no plant species specifically reported for the treatment of the two parasitic, epidemic, local diseases of Chagas (caused by *Trypanosoma cruzi*) and Malaria (Paludism). Only two species, *Cattleya schroederae* Rchb.f. and *Jaccaranda cf. copaia*, were reported for the treatment of Dengue, a viral, epidemic mosquito transmitted disease also affecting these populations (Additional file 3).

**Table 3:** Most commonly treated categories of diseases with the most popular plant species used for their treatments

**Disease category**	**No. of plants reported in both municipalities**	**Popular plant species used in treatments**
Digestive system	35	*Melissa officinalis*
		*Cymbopogon citratus*
		*Apium graveolens*
Infections from bacteria, parasites or fungi	16	*Mentha suaveolens*
		*Citrus limon*
		*Jaccaranda copaia*
Muskuloskeletal system	13	*Verbena littoralis*
		*Urtica dioica*
		*Aristolochia ringens*
Nervous system	10	*Melissa officinalis*
		*Matricaria chamomilla*
		*Lippia citriodora*
Immune system	9	*Aloe vera*
		*Melissa officinalis*
		*Verbena littoralis*

Regarding the plant parts used, leaves were the plant part most frequently reported for remedy preparations, in both municipalities. Other commonly used plant parts were, in descending order of total counts in both municipalities: combination of leaves with stems (36), fruits (16), combination of leaves with branches (9), branches (5), roots (4), and combination of leaves with flowers (3). Other combinations of different plant parts were mentioned only rarely. The use of seeds was not popular in either of the municipalities (Figure 4).

In both municipalities, similar numbers of applications were reported for *Cymbopogon citratus*, *Plantago major*, *Petroselinum crispum* and *Lippia alba* (Mill.) N.E.Br. These species received the highest scores for application of leaves. Most popular species with the highest applications of leaves were: *Cattleya schroederae* (4) and *Piper cf. bogotense* C.DC. (3), in Campo Hermoso, and *Origanum majorana* L. (3) in Zetaquira (Additional file 3).

Eighty different ways of preparation of remedies with medicinal plant species were described. They were grouped according to the type of processing: thermic methods, alcoholic extracts or fresh use. Other ways of preparation of the remedies, such as the combination with food and with other plants (concoction) were also mentioned and thus counted. Practices according to beliefs included the effect of moon or sunlight, esoteric ways and specific ways to treat pets. These additional ways of preparing traditional remedies were included in one group named ‘other’ (Figure 5).

The most popular way of administration remedies was oral followed by the topical in both municipalities (Figure 6). Among the most popular topical ways of administration, ‘baths’ yielded the highest scores (13 counts) in both municipalities, 26 in Campo Hermoso and 25 in Zetaquira. Massage was the second most popular in Campo Hermoso, while ‘emplast’ was the second most popular in Zetaquira.

Healers and amateur healers unanimously reported that bitter plants were denominated ‘hot plants’ (‘plantas calientes’, in Spanish), while plants with sweet flavour were denominated ‘cold plants’ (‘plantas frias’). These terms suggested the level of caution with which the medicinal plants should be used. Bitter plants should be used in small doses at low frequency because most of them can produce eye or heart problems. Bitter plants should be used orally only in situations caused by musculoskeletal complaints. Further, they recommended to initially use topical treatments and, in the most general sense, to follow a diet when using medicinal plants. Popular plants used for the purposes outlined above in the localities are described in Additional file 3.

Table 4 shows the plant species with the highest values obtained in aUV according to the sum of the values from both municipalities. In addition, eUV are also included.

**Table 4:** Plant species according to the highest actual index use values obtained in both municipalities

**Scientific name (Voucher number)**	**aIUV all**	**aIUV Zq**	**aIUV CH**	**eIUV all**	**eIUV Zq**	**eIUV CH**	**Medicinal uses**
*Mentha suaveolens* Ehrh. * (ALCG 127)	1	1.04	0.94	1.10	1.20	0.94	stomach ache(B), headaches(B), intestinal inflammations(B), intestinal parasites(Z), fevers(Z), menstrual cramps(Z), diaper rash(Z)
*Ruta graveolens* L. *(ALCG 86)	0.85	1.08	0.50	1.05	1.24	0.75	against cramps in uterus and menstrual pains(B), rheumatism(Z), intestinal parasites(C), cardio-regulator(Z)
*Melissa officinalis* L. * (ALCG 92)	0.68	0.72	0.63	0.83	0.88	0.75	gripe(B),fevers(B),relaxant(B),stomach ache(B), intestine complaints(B)
*Aloe vera* (L.) Burman f. *˚	0.6	0.63	0.63	0.64	0.64	0.63	asthma (B), cough(B), fever(B), headache(B), healing wounds and skin spots(B), external inflammations(B), hair treatment(B), stomach cancer(Z)
*Ambrosia cumanensis* Kunth (ALCG 63)	0.4	0.81	0.98	0.88	0.88	1.13	gripe(B), rheumatism(B), children bladder complaints(B), menstrual cramps(Z), colds and spams in muscles(C), stomach ache(C), intestinal complaints(C), against flies(Z)
*Lippia alba* (Mill.) N.E.Br. (ALCG 72)	0.56	0.56	0.71	0.68	0.68	0.75	stomach complaints(B), diarrhoea(B), tiredness and weakness(C)/(Z), childbirth(Z)
*Cymbopogon citratus* (DC.) Stapf. * (ALCG 77)	0.6	0.50	0.51	0.56	0.56	0.44	stomach aches(B), intestine complaints(Z), gripe(Z), prostate complaints(C), fevers(C)
*Verbena littoralis* Kunth (ALCG 79)	0.4	0.63	0.93	0.96	0.96	0.88	fevers(B), gripe(B), rheumatism(Z)/ (C), tiredness and weakness(C)
*Urtica dioica* L. * (ALCG 66)	0.32	0.63	0.71	0.44	0.44	1.13	rheumatism(B), allergies(B), blood cleaner and tonic (B), anti-haemorrhagic(B), skin infection and inflammations(C), gripe(C), mouth dryness(C)
*Citrus limon* (L.) Burm. F. * (ALCG 93)	0.2	0.63	0.71	0.52	0.52	1.00	gripe(B), fevers(B), infections in throat(B), stomach and intestine complaints(B), rheumatism(C), eczema (C), headache(C), to prevent high blood pressure(Z)
*Jacaranda cf. copaia* (Aubl.) D.Don (ALCG 69)	0.12	0.69	0.49	0.2	0.2	0.94	Intestinal complaints(B), Kidney complaints(Z), rheumatism(C), gripe(C), fevers(C), dengue(C), skin infections(C), circulatory complaints(C), mouth dryness(C)
*Matricaria chamomilla* L. *(ALCG 96)	0.32	0.38	0.34	0.32	0.32	0.38	stomach-complaints(B),relaxant(B), rheumatism(Z),colds, spams and cramps in muscles (C)
*Rosmarinus officinalis* L. *(ALCG 149)	0.44	0.19	0.39	0.44	0.44	0.31	lungs complaints(B), tooth ache(B), hypertension complaints(B), against hair loss(B), muscles pain (C) rheumatism(C), stomach ache(Z), tiredness(Z)
*Petroselinum crispum* (Mill. Nyman ex A.W. Hill*(ALCG 87)	0.36	0.25	0.46	0.44	0.44	0.50	cardiovascular complaints as high blood pressure(B), enhance stomach function(B), halitosis(B), condiment herb(B), kidney complaints(C), intestinal pain(C), against menstrual pain(Z)
*Lippia citriodora* (Lam.) Kunth (ALCG70)	0.36	0.13	0.39	0.48	0.48	0.25	stomach and intestinal complaints(B), relaxant(B), general indisposition(Z)
*Mentha viridis* L. * (ALCG 91)	0.2	0.38	0.41	0.36	0.36	0.50	gripe(B), stomach ache(B), inflammations of intestine(B)
*Sambucus nigra* L. *(ALCG 68)	0.24	0.25	0.66	0.72	0.72	0.56	gripe(B), eye inflammations and infections(B), fevers(B), tiredness and weakness(Z), respiratory, complaints(B), head ache(Z), against hair lice(C)
*Cattleya schroederae* Rchb.f. (ALCG 165)	0	0.63	0.27	0	0	0.69	typhoid(C), dengue(C), fever(C), intestinal complaints(C)
*Calendula officinalis* L. * (ALCG 57)	0.24	0.19	0.49	0.6	0.6	0.31	external-internal inflammations(B), gastritis(C), skin infections (Z), menstrual cramps(Z)
*Ocimum campechianum* Mill. (ALCG 74)	0.16	0.31	0.41	0.28	0.28	0.63	intestinal complaints(B), eye dust (B), gripe(Z)
*Chenopodium ambrosioides* L. (ALCG 80)	0.12	0.38	0.41	0.4	0.4	0.44	stomach complaints(B), intestinal parasites(B)
*Citrus maxima* (Burm. ex Rumph.) Merr. *(ALCG 98)	0.24	0.19	0.32	0.36	0.36	0.25	gripe(B), stomach ache(B), inflammations because infections(C), headache(Z), fevers(Z), rheumatism(Z)
*Apium graveolens* L. * ˚	0.24	0.19	0.22	0.2	0.2	0.25	intestinal obstipation(B), stomach ache(B), condiment herb(B), relaxant(B), to regulate menstruation(Z)
*Plantago major* L*.(ALCG 374)	0.16	0.31	0.51	0.32	0.32	0.81	eye dust and infections(B), gastritis(B), liver cleanser(B), kidney complaints(C), fever(C), eczema(C), healing wounds(Z)
*Citrus aurantium* var. *amara* L. *˚	0.2	0.25	0.39	0.2	0.2	0.69	relaxant(B), headache(C), mouth infections(B), rheumatism(B), body dryness(Z)

Includes also estimated index use values.

*Introduced plant species; ˚No specimen collected; (C) Campo Hermoso; (Z) Zetaquira; aIUV Actual Index Use Value; eIUV Estimated Index Use Value; all-aIUV/eIUV Sum of all values corresponding to actual or estimated uses.

### Differences and similarities among plant groups and families

UVs of introduced plant species were significantly higher than native species in the more accessible municipality of Zetaquira (Table 5), while there were no significant differences between the two groups in the more remote municipality of Campo Hermoso and nor in the two municipalities combined.

**Table 5:** Results of Mann–Whitney-Wilcoxon tests of differences in Index Use Values (IUV) among native and introduced medicinal plants

	**Campo Hermoso**			**Zetaquira**			**Both Municipalities**		
**Index**	**Med. Nat.**	**Med. Int.**	**P**	**Med. Nat.**	**Med. Int.**	**P**	**Med. Nat.**	**Med. Int.**	**P**
Actual IUV	0.13	0.13	0.677	0.04	0.14	0.005	0.09	0.12	0.095
Estimated IUV	0.25	0.25	0.750	0.12	0.20	0.047	0.22	0.23	0.237

Med. = Median; Nat. = Native; Int. = Introduced.

Numbers of plant species for which estimated and actual UVs could be calculated did not differ greatly between the two municipalities (Table 6). Nevertheless, totals of actual UV of introduced species were higher in Zetaquira than in Campo Hermoso. Totals of actual UV of native species were higher in Campo Hermoso than in Zetaquira. Totals of common plant species including introduced and native ones were very high in both municipalities.

**Table 6:** Numbers of introduced and native medicinal plant species for which estimated and actual index use values could be calculated

**Origin**	**Index use value**	**Campo Hermoso**	**Zetaquira**	**Both**
Introduced	Actual	4	10	25
	Estimated	2	3	38
Native	Actual	11	5	18
	Estimated	7	2	26

There were many similarities in the use of both introduced and native plant species between the municipalities (Table 7).

**Table 7:** Jaccard-index similarity (%) between Campo Hermoso and Zetaquira municipalities for medicinal plant species with estimated and actual index use values

**Index use value**	**Introduced**	**Native**
Estimated	88	74
Actual	64	53

Estimated Family Use Values (FUV) were higher than actual family use values in almost all cases (Table 8). Estimated and actual FUV were commonly higher for Campo Hermoso than for Zetaquira. Plant families represented by high numbers of plant species did not always have high FUVs, i.e. Asteraceae and Apiaceae. Some families, of which only one plant species was reported, surprisingly obtained high values, e.g. Aristolochiaceae which had the highest estimated use value. Another interesting case was the Asphodelaceae which yielded high actual use values. Furthermore, Asphodelaceae and Poaceae coincided in having high actual use values in both municipalities.

**Table 8:** Family Use Values (FUV) of most popular plant families calculated from estimated and actual index use values of species and number of native and introduced species per family

**Family**	**Native**	**Introd.**	**FUV est. CH**	**FUV est. Zq**	**FUV est. Both**	**FUV act. CH**	**FUV act. Zq**	**FUV act. Both**
**Asteraceae**	4	6	0.26	0.28	0.27	0.16	0.16	0.16
**Lamiaceae**	2	5	0.48	0.59	0.55	0.36	0.43	0.40
**Apiaceae**	1	3	0.22	0.20	0.21	0.14	0.19	0.17
**Rutaceae**	0	4	0.67	0.58	0.62	0.39	0.43	0.41
**Verbenaceae**	4	0	0.53	0.58	0.56	0.39	0.35	0.37
**Malvaceae**	0	3	0.29	0.07	0.15	0.10	0.04	0.07
**Solanaceae**	1	2	0.29	0.15	0.20	0.08	0.05	0.07
**Urticaceae**	2	1	0.48	0.24	0.33	0.23	0.13	0.17
Acanthaceae	2	0	0.13	0.40	0.30	0.00	0.12	0.08
Alliaceae	0	2	0.22	0.22	0.22	0.13	0.06	0.09
Amaranthaceae	1	1	0.44	0.28	0.34	0.31	0.14	0.21
Bignoniaceae	2	0	0.50	0.10	0.26	0.38	0.06	0.18
Commelinaceae	1	1	0.09	0.08	0.09	0.06	0.04	0.05
Myrtaceae	1	1	0.44	0.34	0.38	0.25	0.04	0.12
Phytolaccaceae	2	0	0.31	0.04	0.15	0.25	0.00	0.10
Piperaceae	2	0	0.47	0.04	0.21	0.31	0.00	0.12
Adoxaceae	0	1	0.56	0.72	0.66	0.25	0.24	0.24
Aristolochiaceae	0	1	0.75	0.64	0.68	0.00	0.04	0.02
Asphodelaceae	0	1	0.63	0.64	0.63	0.63	0.60	0.61
Plantaginaceae	0	1	0.81	0.32	0.51	0.31	0.16	0.22
Poaceae	0	1	0.44	0.56	0.51	0.50	0.60	0.56

Bold = plant families with higher number of species (>2); italics = plant families with two plant species; underlined = plant families with one species but high FUV. Est. = estimated; act. = actual; CH = Campo Hermoso; Zq = Zetaquira; both = both municipalities together.

The list of the most popular medicinal plant species based on median of actual and estimated Use Values, among amateur healers and healers is shown in Table 9.

**Table 9:** Most popular species among healers and amateur healers

**Amateur healers**	**Healers**
*Calendula officinalis* L.	*Jacaranda copaia* (Aubl.) D. Don
*Citrus Limon (*L.) Burm.F	*Aristolochia ringens* Vahl.
*Petroselinum crispum (*Mill.) Nyman ex A.W. Hill	*Ruta graveolens* L.
*Chenopodium ambrosioides* L.	*Lippia alba* (Mill) N.E. Brown
*Ruta graveolens* L.	*Althernanthera lanceolata* Benth.
*Cymbopogon citratus* (DC.) Stapf	*Psidium guinense* Sw*.*
*Mentha suaveolens* Ehrh.	*Rubus glaucus* Benth*.*
*Allium sativum* L.	

From the amateur healers’ list, 87% species were introduced including six herbs and one shrub, while only 13%, i.e. one herb, was native. In the case of healers, 57% in the list of preferred species were native, which included two trees and two shrubs, while the remaining 43% were introduced, i.e. two herbs and one vine (Additional file 3).

## Discussion

### Significance of the use of medicinal plants within the municipalities

The patterns in traditional knowledge and medicinal plant use described in this study is in accordance with other studies [25-27] in the high number of introduced species mostly herbs. Especially for the more accessible municipality of Zetaquira the use of native species was rarely reported.

Rapid adoption of introduced species in traditional medicine can be understood as a response to new opportunities arising with globalisation. Inefficacy of native species may lead to experimentation with introduced species. Introduced species enrich the arsenal of species used in treatments and are often seen as very powerful [28-30]. Adoption of introduced species may be seen as a way to reshape and re-vitalise traditional practices, which in many places provide an important alternative to the official health care services within developing countries.

The effectiveness of treatments with popular introduced plant species that are used world-wide indicates the need to promote and further the studies on the use and effectiveness of these plant species [23,24]. In the Colombian pharmacopoeia list of officially accepted plants for medicinal use 44% of the total of 149 species are introduced [23,31].

The flip-side of the coin is that with the adoption of introduced medicinal plant species peoples’ interest in cultivating native species decreases. In this study most of the popular medicinal plant species (UV >0.5) were introduced with the exception of *Verbena littoralis* and *Ambrosia cumanensis* (Table 4, Additional file 3). Furthermore, a number of native species with multiple uses, e.g. timber but also medicinally, are currently endangered or have disappeared from the regions indicating that over- exploitation has taken place [27].

The high similarities between estimated and/or actual UV (> 50%) for introduced and native plant species showed little difference in the lists of medicinal plants between the municipalities. For introduced species, similarity values were the highest (Table 7). These results are comparable to the findings of De Ameida et al. [28] who worked with rural communities in the Northeast Brazil.

Overexploitation of medicinal plant species, including a large number of native species, has been discussed extensively. Njoroge et al. [32] found that *Carissa edulis* (Forssk.) Vahl, a native wild species of Ethiopia [33], is a priority species used in the treatment of several ailments, especially for stomach pains, a common disorder in the Mwingi District, Kenya. Presently, this species is threatened by overuse. Similarly, other scholars have reported that plant species most popularly used by communities depend on the kind of local diseases that people face [2], which could be a parameter to identify possible species that could become endangered by overexploitation in combination with knowledge on plant parts used, harvest techniques, demand and prices.

The most frequent diseases in the study sites according to local reports from the health centre service [34] were related to the digestive system and infections caused by bacteria, parasites or fungi. The present study shows that the highest valued species were related to exactly these diseases. Of these, the top five were all introduced (Additional file 3 and Table 4) confirming that communities make use of specific plant species including introduced ones.

The large number of plant species used in the treatment of complaints of the digestive system, infections and nervous system in both municipalities, is comparable to other findings from studies involving farmer communities in other localities in department of Boyacá. For example, Lagos [9] found that stomach-complaints occupy the category of illnesses which is treated with the highest number of medicinal plant species, followed by complaints of the nervous system. At the same time, these results are comparable to findings from other places. In the municipalities of the city of Imbituba, Santa Catarina, Brazil Zank and Hanazaki [35] found, that digestive complaints were the disease category with the highest therapeutic applications of medicinal plants. Furthermore, Neves et al. [36], who studied various groups of the community of Trás-os-Montes, Portugal, including farmers, reported that illnesses related to the digestive system were most popularly treated with medicinal plants.

### Life form, habitat and proximity to cities facilitate popularity of introduced species

It has been demonstrated in several studies [28,37,38], that life form and habitat specification determine the use of medicinal plant species. The reason usually mentioned is that herbs, of which a large number are introduced, are easy to cultivate and maintain in small gardens or pots near to or in the houses. Examples of species whose popularity can be related to life form and the ease of cultivation are *Ruta graveolens* that has a high medicinal value in Zetaquira, and *Urtica dioica* that is a highly valuable herb in Campo Hermoso. Opposed to the introduction of easily cultivated herbs, a number of ecological factors diminish advantages of cultivation and maintenance of some native plant species.

Among the most popular species of amateur healers (eight species), seven species (87.5%) were introduced, of which six were herbs and only one a shrub (Table 9). In contrast, among healers the tendency to use native species, usually trees or shrubs, is higher: 57% of their most popular species was native and included two herbs and one vine. Since healers are specialised in the use of medicinal plants, they are in most cases willing to cultivate a diversity of species, including trees and shrubs with medicinal values, in their home gardens and on other cultivated lands. Hence, cultivation could explain the popularity of native woody species within the group of healers.

The high significance of introduced plant species according to UV (Table 5) in Zetaquira closer to the capital of the department, Tunja, could be an indication of the influence of distance to modern cities and acculturation upon the adoption of new medicinal plant species. These findings are comparable to results obtained by other studies, e.g. in Brazil, Argentina and Manus Island (Papua New Guinea) [28,39,40]. Inhabitants of Campo Hermoso, which is more remote and follows rural traditions, have higher knowledge on medicinal plant use. Furthermore, there are more healers in Campo Hermoso. Hence, the null hypothesis of similarity between plant knowledge and use between municipalities is falsified.

#### High presence of pharmacologic components in herbs and popularity of genera

The high presence of pharmacologic components in herbs makes them attractive for treatments of different diseases [27]. Specifically the high composition of alkaloids in leaves facilitates medicinal uses as has been indicated by the popularity of these plant parts within several communities [9,25,27,36] and also in the present study. Furthermore, a number of phytochemical studies have proven remarkable alkaloid and oil content in herbal leaves [41-43] which may provide alternatives for the pharmaceutical industry. Taxa with large numbers of useful herbs contribute to the tendency of using leaves and introduced plant species. For example, the genus *Mentha*, one of the most popular taxa in Campo Hermoso and Zetaquira and, was reported to be used in treatments of digestive complaints, colds, fevers, skin infections, inflammations and headaches (Additional file 3). *Mentha ssp.*, introduced to the American continent, is widely popular in folk medicine [9,35,44] and contribute to the popularity of introduced plant species.

### Popularity of plant families

Popularity may also reflect world-wide presence of large plant families, such as the Lamiaceae. Contrary, Schippmann et al. [1], suggest that popularity of plant families is related to the local availability of the species. Studies in diverse localities as for example in Loja province, Ecuador [45], Imbituba, Santa Catarina, Brazil [35], Sierras de Córdoba, Argentina [46], and Banbungo, Cameroon [27] have found Asteraceae and Lamiaceae with high numbers of plant species used locally. These findings are comparatively similar to the results obtained by ethnobotanical studies carried out in the department of Boyacá, e.g. the studies of Lagos [9] in five municipalities in the central part of Boyacá, and a study carried out in the municipality of San José de Paré by Toscano [10]. The results indicate a large availability of species belonging to the Asteraceae and Lamiaceae in this region. At the same time, these results are comparable to the global pattern suggested by Moerman [47] who considered that Asteraceae and Lamiaceae are among the most used plant families in traditional medicine world-wide.

In relation to the popularity of Asteraceae, Garcia Barriga [48] reported that this family is the taxonomic group of plant species with the highest use in traditional medicine in Colombia. Nevertheless, the large range of distribution of this family may explain part of the popularity of this taxonomic group in folk medicine [27].

Furthermore, plant families, such as the Apiaceae, Apocynaceae and Guttiferae, of which there are examples of species with medicinal use in Campo Hermoso and Zetaquira, are part of the list of plant families with high numbers of traditionally used species worldwide [1]*.*

However, 85% of the medicinal species identified in this study have been described and reported as medicinal in Colombia [11,48-50]. Of these species, 54% are introduced. This indicates that transmission of knowledge on introduced medicinal species has been successful in Colombia and in the studied municipalities. Similar situations have been observed and discussed in other countries as Brazil [28] where it was observed that knowledge of introduced plant species within farmers communities has been well established.

Promotion, conservation and sustainable use of native medicinal plant species may be facilitated by including shrubs and trees into local agroforestry systems. A number of these species, apart from being medicinal, offer also other kind of uses, e.g. as food, source of wood and shade. Such multiuse plants are often valuable to locals [31].

## Conclusions

A total of 80 medicinal plant species were recorded. Of these, 78 species were taxonomically identified of which 35 were native species and 43 were introduced belonging to 74 genera and 41 floristic families. Among the native species 40% were found in natural habitats while twenty-seven per cent of introduced species were found as naturalised in the wild. The families with the highest numbers of species reported as medicinal were: Asteraceae*,* Lamiaceae*,* Apiaceae*,* Rutaceae, Verbenaceae, Malvaceae*,* Solanaceae and Urticaceae. Most applications of medicinal plants are related to diseases of the digestive and respiratory systems, and infections. Leaves are the most popular plant part used. Decoctions and oral administration are the most common practices.

The total of medicinal plants uses described by informants was higher in the more remote Campo Hermoso than in the more accessible Zetaquira. UVs of introduced plant species were significantly higher than native species in the more accessible municipality of Zetaquira, while there were no significant differences between the two groups in the more remote municipality of Campo Hermoso and nor in the two municipalities combined.

The list of the most popular medicinal plant species for healers and amateur healers respectively showed that 87% of the species from the amateur healers’ list were introduced including mainly herbs while in the case of healers, 57% of the species of mainly trees and shrubs. Conversion of forests for agriculture and cattle ranching is depleting local forest resources and a number of medicinal plants were reported as disappearing or locally extinct. Only about 50% of the native medicinal species were found in cultivation. Agroforestry may provide an option for integrating agriculture with cultivation of native trees, shrubs and lianas, not otherwise cultivated. Educational programs could raise awareness in relation to conservation and maintaining use of native species.

## Consent

Written informed consent was obtained from the patient for publication of this report and any accompanying images.

## Endnotes

^a^ Group of experts on the use of local medicinal plant species who offer local people treatments using medicinal plants.

^b^ Group of people who are interested in the use of medicinal plants, but do not work as professional healers.

^c^ Local term to refer to large home gardens with inclusion of a variety of trees and shrubs.

## Competing interests

The authors declare that they have no competing interests.

## Authors’ contributions

Ana Lucia Cadena-González was the main responsible for study design, data analysis, interpretation, and writing. She conducted the field work in Campo Hermoso and Zetaquira between October 2008 and February 2009, collected the plant specimens, and identified the specimens in cooperation with the herbaria. Marten Sørensen and Ida Theilade contributed to the concept and design of the study, interpretation of the findings and preparation of the manuscript. All authors read and approved the final manuscript.

## Supplementary Material

Additional file 1 **Categories of diseases.** Description of the 14 categories of diseases recorded from informants in the municipalities of Campo Hermoso and Zetaquira. Data includes the list of the diseases within each category. Click here for file

Additional file 2 **Spearman rank correlations.** Spearman rank correlations of different variants of knowledge indices. Correlation coefficients are given above the diagonal; corresponding p-values below the diagonal. Click here for file

Additional file 3 **Ethnobotanical descriptions of medicinal plant species.** List of medicinal plant species reported in Campo Hermoso and Zetaquira including ethnobotanical characteristics. Data provided included: Taxonomical family, scientific name, vernacular names, voucher ID, life form, habitat type/place of collection, place of origin, estimated and actual Index Use Values for both municipalities, plant part used, use, preparation and mode of administration. Additionally, species reported in the Colombian Vademecum (2008) and in WHO monographs (2009) are indicated, as well the plant material of interest reported in these documents that was also reported within the municipalities. Species reported as endangered from the perception of the locals within the municipalities are also noticed. Click here for file
