# Supplementary material for: Use and valuation of native and introduced medicinal plant species in Campo Hermoso and Zetaquira, Boyacá, Colombia
Source: J Ethnobiol Ethnomed. 2013 Apr 11;9:23. doi: 10.1186/1746-4269-9-23 (PMC3641003; doi:10.1186/1746-4269-9-23)
Supplement: Additional file 3 — Ethnobotanical descriptions of medicinal plant species. List of medicinal plant species reported in Campo Hermoso and Zetaquira including ethnobotanical characteristics. Data provided included: Taxonomical family, scientific name, vernacular names, voucher ID, life form, habitat type/place of collection, place of origin, estimated and actual Index Use Values for both municipalities, plant part used, use, preparation and mode of administration. Additionally, species reported in the Colombian Vademecum (2008) and in WHO monographs (2009) are indicated, as well the plant material of interest reported in these documents that was also reported within the municipalities. Species reported as endangered from the perception of the locals within the municipalities are also noticed. [file 1746-4269-9-23-S3.doc]

| Additional file 2: List of medicinal plant species reported in Zetaquira and Campo Hermoso including ethnobotanical characteristics | | | | | | | | | |
| --- | --- | --- | --- | --- | --- | --- | --- | --- | --- |
|  |  |  | Index Use Values (estim./ actual) | | |  | |  |  |
| Species, family,  voucher ID,  and vernacular names | Life form,  Habitat/ place of collection | Status,  place of Origin | Zeta-quira | Campo Herm. | Part Used | | Use | | Preparation, mode of administration |
| *Justicia filibracteolata* Lindau  Acanthaceae  ALCG 62  Chuchuhuaza | shrub  cultivated in homegarden | native  Colombian Andes^3^ | 0.48/ 0.04 | 0.06/ 0.00 | bark and leaves (B) | | rheumatism (B) | | concoction with Allium sativum and Aristolochia ringens in extract in alcohol/ oral -U1- (Z), concoction with Cannabis sativa  in extract in alcohol -U2-/ oral (B), decoction/ oral (C) |
| *Trichanthera gigantea* (Bonpl.) Nees (s)  Acanthaceae  ALCG 125  Cafetero, Quiebrabarrigo | small tree  wild in edge of route | native to Northern South  America^8^ | 0.32/ 0.20 | 0.19/ 0.00 | leaves and stems (B) | | inflammations and skin infections (B)  fevers (Z)  to lose weight (Z) | | decoction/ topical-baths (B)  decoction/ topical-baths (Z); fresh extract by maceration/ oral (Z)  infusion/ oral (Z) |
| *Sambucus nigra* L.* º  Adoxaceae•  Caprifoliaceae ^++^  ALCG 68  Sauco  Elder or Elderberry | tree  cultivated in  solar | introduced  Europe-West Siberia-North Africa ^5^ | 0.72/ 0.24 | 0.56/ 0.25 | leaves and flowers (B)*^2^  flowers (B)º ^2^  leaves and branches (B)  flowers and shoots (Z)  leaves and branches (B) | | gripe (B)  eye inflammations and infections (B)  fevers (B)  tiredness and weakness (Z)  respiratory complaints (B)  respiratory complaints (Z)  headache (Z)  against hair lice (C) | | concoction with *Citrus limon* juice in decoction/ oral (B)  extract in decoction or destilation (in a glass bottle)/ topical- eye baths (B) decoction/ topical in baths (B)  decoction/ topical, baths (Z)  decoction/ oral (B)  concoction with milk in decoction/ oral (Z)  decoction/ oral (B)  decoction/ oral (Z)  fresh extract from maceration/ topical on the head before the sunrise starts (Z)  fresh extract from maceration/ topical for washing the hair (C) |
| *Furcraea macrophylla* Baker, Hook (e-CH)  Agavaceae  Fique,cabuya  Fique | succulent  rosette  forming  tall herb  cultivated in  a farmland | native  Boyaca-Colombia^7^ | 0.00/ 0.00 | 0.25/ 0.13 | root (C)  leaves (C) | | rheumatism (C)  muscles and tendons complaints (C)  Hidropisy (C) | | decoction/ oral (C)  use of fibres of leaves to tie them on the affected part/ topical (C)  decoction of a piece of leaf/ oral (C) |
| *Chenopodium ambrosioides* L. (s)  Amaranthaceae  ALCG 80  Paico  Epazote  Wormseed | herb  ascendent  cultivated  in small  garden | native  Central Andean range and south of western range in Colombia ^7^ | 0.40/ 0.12 | 0.44/ 0.38 | leaves (B)  leaves and stems (B) | | stomach complaints (B)  intestinal parasites (B) | | infusion or decoction/ oral (B)  fresh extract by maceration in water/ oral (B) |
| *Althernanthera lanceolata* (Benth.) Schinz  Amaranthaceae  ALCG 113  Planta del cáncer or Sanguinaria | herb  cultivated in  small garden | introduced  American tropics and subtropics, Asia, Africa and Australia^12^ | 0.16/ 0.16 | 0.48/ 0.25 | leaves and branches (B)  leaves (C)  leaves (Z) | | healing of wounds (B)  skin cancer (C)  stomach ulcer (C)  cancer in general (C)  cancer in general (Z)  fevers (Z) | | decoction/ oral and decoction/ topical-baths (B)  decoction/ topical baths (C)  decoction in milk/ oral (C)  decoction / oral (C)  fresh extract by maceration in water/ oral (Z) |
| *Eryngium foetidum* L.*  Apiaceae  ALCG 167  Cilantrón,cilantro cimarrón  Mexican coriander, shadow beni | herb  cultivated in  homegarden | native  South Mexico to Bolivia Colombian Andeans and Orinoquia^5^ | 0.00/ 0.00 | 0.06/ 0.06 | root (C)  leaves (C)*2 | | hepatitis (C)  condiment herb (C) | | decoction/ oral (C)  combination with food/ oral (C) |
| *Apium graveolens* L. *  Apiaceae  Apio  Celery,celeriac | herb  cultivated in  homegarden | introduced  Europe and West Asia ^13^, Europe ^5^ | 0.20/ 0.24 | 0.25/ 0.19 | leaves and stems (B)*2 | | intestinal obstipation (B)  stomach ache (B)  condiment herb (B)  relaxant (B)  to regulate menstruation (Z) | | decoction/oral(B)  infusion/oral(B)  combination with food/oral(B)  infusion/oral(B)  decoction/oral(Z) |
| *Foeniculum vulgare* Mill.* º  Apiaceae  ALCG 89  Hinojo  Fennel, Bronze Fennel,  Sweet cumin^12^ | herb  cultivated in  homegarden | introduced  Mediterranean Europe^5^ | 0.16/ 0.16 | 0.06/ 0.06 | leaves and  stems (B) | | to enhance lactation (B)  condiment herb (B) | | decoction in milk or "aguadepanela"-U3-/ oral (B)  combination with food/ oral (B) |
| *Petroselinum crispum* (Mill.) Nyman ex A.W. Hill*  Apiaceae  ALCG 87  Perejil  Parsley | herb  cultivated in  homegarden | introduced  Eastern Mediterranean Western Asia Mediterranean Europe ^8^ | 0.44/ 0.36 | 0.50/ 0.25 | leaves (B)*2 | | cardiovascular complaints as high blood pressure (B)  enhance stomach function (B)  againts bad breath (B)  condiment herb (B)  kidney complaints (C)  intestinal pain (C)  againts menstrual pain (Z) | | infusion/ oral (B)  to chew leaves/ oral (B)  combined with food/ oral (B)  infusion/ oral (C)  infusion/ oral (Z) |
| *Catharanthus roseus* (L.) G. Don.  Apocynaceae  ALCG 158  Vicaria  Madagascar periwinkle | short lived  herb  cultivated in  pod | introduced  Madagaskar ^8^ | 0.00/ 0.00 | 0.06/ 0.06 | flowers (C) | | againts eye infections and irritations (C) | | infusion/ topical-baths (C) |
| *Philodendron* sp.  Araceae  ALCG 103  Hidra or Yedra | sufrutex  wild in edges  of secundary  forest | unknown  Africa and Asia and America around the equador ^7^ | 0.04/ 0.04 | 0.00/ 0.00 | leaves and stem (Z) | | skin infections and inflamations (Z) | | fresh extract by maceration/ topical-rub part affected |
| *Colocasia esculenta* (L.) Schott  Araceae  ALCG 106  Malanga  Taro | herb  cultivated in  homegarden | introduced  South East Asia,  Australia ^8,13^ | 0.20/ 0.16 | 0.31/ 0.25 | tuberous  root (B) | | gastritis (B)  stomach ulcer (B)  food (B) | | concoction of blended root with milk/ oral (B)  carbohydrate source food/ oral (B) |
| *Aristolochia ringens* Vahl.  Aristolochiaceae  ALCG 78  Bejuco de Guaco  Gaping Dutchman`s Pipe | Twiner/  climber  Wild in  river bank | introduced  native to  Brazil ^15^ | 0.64/ 0.04 | 0.75/ 0.00 | leaves and stems (B) | | rheumatism (B)  ofidic accidents (B) | | concoction with Cannabis sativa, Erythroxylum coca and Justicia filibracteolata in extract in alcohol/ topical and oral (B), maceration in extract in alcohol/ topical and oral (Z)-U2-  maceration/ topical in emplast (B) |
| *Aloe vera* (L.) Burman f. * º  Asphodelaceae  Zábila  Aloe | Short stem-  med suculent  plant  cultivated in  pods | introduced  arabic peninsula ^8^ | 0.64/ 0.60 | 0.63/ 0.63 | gel from leaves (B)  * 2 º 2 | | asthma (B)  caugh (B)  fever (B)  headache (B)  healing wounds and skin spots (B)  external inflamations (B)  hair treatment (B)  stomach cancer (Z) | | fresh gel is blended single or in concoction with egg or with honey and juice of Citrus limon/ oral (B)  fresh gel/ topical-emplast over head-front (Z)  fresh gel/ topical-emplast over head-front (B)  fresh gel/ topical-emplast or for massaging (B)  fresh gel in concoction with honey/ oral (Z) |
| *Bidens pilosa* L. *  Asteraceae  ALCG 144  Chipaca  Spanish needle,black jack | herb  wild in pastures  with bushes  composition | native  From the centrum to the south of the Andeans of Colombia^3^ | 0.00/ 0.04 | 0.00/ 0.00 | leaves (Z)*2 | | skin infections (Z) rashes (Z) | | maceration/ topical-emplast (Z)  decoction/ topical-baths (Z) |
| *Onoseris onoseroides* (Kunth) Robins  Asteraceae  ALCG 139  Santa María | tall herb  wild in slope hills and between bushes on open pastures | introduced  Central America and naturalized in Colombia ^3^ | 0.00/ 0.00 | 0.13/ 0.00 | leaves (B) | | against bleedings (C)  against uterus complaints (C)  pains in muscles (Z) | | fresh leaves directly on the wound/ topical (C)  infusion/ oral (C)  maceration/ topical-emplast (Z) |
| *Conyza filaginoides* (DC.) Hieron  Asteraceae  ALCG 110  Venadillo | herb  spontaneous in solar with some cultivated trees | native  Central Colombian Andeans ^3^ | 0.04/ 0.00 | 0.00/ 0.00 | leaves and stems (Z) | | skin infections and  rash (Z) | | fresh extract by maceration/ topical-massage (Z) |
| *Pyrethrum parthenium* (L.) Sw.  * º  Asteraceae  ALCG 75  Manzanilla  Feverfew  Wild chamomille | herb  cultivated in  small garden | introduced  Eurasian, meridional-boreal^3^ | 0.36/ 0.28 | 0.00/ 0.00 | leaves º2*2 and stems (Z)*2  flowers (Z) | | intestinal complaints (Z)  relaxant (Z)  uterus cramps (Z) | | infusion/ oral (Z)  fresh maceration/ emplast (Z) |
| *Galinsoga parviflora* Cav.  Asteraceae  ALCG 90  Guacas | herb  cultivated in  homegarden | native  Colombia,  South  America^3,8^ | 0.08/ 0.04 | 0.19/ 0.06 | leaves (B)  leaves and stems (C) | | againts excess of stomach acids (B)  condiment herb (B)  against white plaques in mouth (C)  intestinal parasits (C) | | decoction/ oral (B)  decoction/ oral (B)  fresh extract by maceration in water/ oral (C) |
| *Matricaria chamomilla* L. * º  Asteraceae  ALCG 96  Manzanilla dulce  Roman chamomile,  German chamomile | herb  cultivated in  homegarden | introduced  Iran and Europe ^3,8,17^ | 0.32/ 0.32 | 0.38/ 0.38 | leaves and flowers (B)  leaves, stems and flowers (B) | | stomach complaints (B)  relaxant (B)  rheumatism (Z)  colds, spams and cramps in muscles (C) | | decoction or infusion (B)  infusion/ oral (B)  fresh extract by maceration/ topical-massage (Z)  decoction/ oral and fresh extract by maceration/ topical-massage (C) |
| *Taraxacum officinales* F.H.Wigg.  * º  Asteraceae  ALCG 104  Diente de león  Dandelion | herb  spontaneous in  a farm with  a large patch of secondary forest | introduced  Europe^3^ | 0.16/ 0.12 | 0.25/ 0.06 | leaves (B)  * 2 | | cardiovascular complaints (B)  kidney complaints (C)  enhance lungs function (Z)  rheumatism (Z)  liver cleanse (Z) | | infusion/ oral (B)  infusion/ oral (C)  infusion/ oral (Z)  decoction/ oral (Z)  maceration/ topical-emplast (Z)  infusion/ oral (Z) |
| *Artemisia absinthium* L. *  (e-CH)  Asteraceae  Ajenjo  Absinthe wormwood,  grand wormwood | herb  cultivated in  homegarden | introduced  Europe^3^ and West Asia | 0.32/ 0.16 | 0.19/ 0.13 | leaves (B)  leaves (Z)  leaves and stems (Z) | | stomach ache (B)  liver cleanser (C)  to prevent extra  blood flow during child delivery (Z)  againts inflamations and infections (Z) | | infusion/ oral (B)  infusion/ oral (C)  infusion/ oral (Z)  infusion/ oral (Z)  decoction/ topical-baths (Z) |
| *Calendula officinalis* L. * º  Asteraceae  ALCG 57  Caléndula  Pot Marigold,  Scotch Marigold | short-lived plant  cultivated in pod | introduced  South and East Europe^3^ | 0.60/ 0.24 | 0.31/ 0.19 | flowers (B)  *2 º 2  leaves and flowers (C)  leaves and stems (Z) | | external-internal inflamations (B)  gastritis (C)  skin infections (Z)  menstrual cramps (Z) | | Decoction / topical, baths (B)  decoction / oral (B)  infusion of leaves and flowers in concoction with drops of Crotons' sage/ oral (C)  maceration of leaves/ topical (Z)  decoction/ topical,baths (Z) |
| *Ambrosia cumanensis* Kunth  Asteraceae  ALCG 63  Altamisa  Western Ragweed or Perennial Ragweed | herb  cultivated in  homegarden | native  Colombian Andeans^3^ | 0.88/ 0.40 | 1.12/ 0.81 | leaves and stems (B)  branches (Z) | | gripe (B)  rheumatism (B)  children  bledder complaints (B)  menstrual cramps (Z)  colds and spams  in muscles (C)  stomach ache (C)  intestinal complaints (C)  against flies (Z ) | | infusion/ oral (B)  fresh plant in maceration / topical in cataplasm (B)  concoction with Mentha suaveolens and Ruta graveolens in infusion/ oral (Z)  fresh extract by maceration/ topical to rub body (C)  decoction/ oral (C)  branches are placed in dogs beds against flies (Z) |
| *Anredera cordifolia* Tenore  Basellaceae  ALCG 109  Rubacá | Vine  wild in edges  of secondary forest  with enought  shadow and  moisture | native  SouthAmerica (Colombia,Ecua-dor, Argentina, Paraguay, Uruguay, Bolivia,Brasil,Salvador)^13^ | 0.24/ 0.16 | 0.12/ 0.13 | leaves and stems (B) | | fevers (B)  respiratory complaints (C)  skin infections (Z)  muscles and tendons inflamations,edemas (Z) | | decoction or infusion/ oral (B)  fresh extract by maceration in water/ topical-baths (Z)  decoction/ oral (C)  fresh extract by maceration/ topical-emplast or massage (Z) |
| *Crescentia cujete* L. ^3^,; *  Bignoniaceae  Totumo  Calabash tree | tree  cultivated in  homegarden | native  North Central America, Mexico and Belice, Colombian Andes,Amazons and Caribean^3^ | 0.00/ 0.00 | 0.06/ 0.06 | young fruit (C)*2 | | caugh (C)  asthma (C) | | fresh extract or juice/ oral (C) |
| *Jacaranda cf. copaia* (Aubl.) D.Don  Bignoniaceae  ALCG 69  Gualanday | tree  cultivated in  solar | native  Colombian Andeans and Orinoquia^3^ | 0.20/ 0.12 | 0.94/ 0.69 | bark (B)  leaves (Z)  leaves (C)  branches (C) | | Intestinal complaints (B)  Kidney complaints (Z)  rheumatism (C)  gripe (C)  fevers (C)  denge (C)  skin infections (C)  circulatory complaints (C)  mouth dryness (C) | | decoction/ oral (B)  infusion/ oral (Z)  infusion/ oral (C)  concoction with fresh maceration-Eucalyptus globulus and Allium sativum in extract in alcoholU2 / oral  topical (C)  decoction/ oral (C)  see concoction with Urtica dioica and Rosmarinus officinalis (C) |
| *Symphytum officinale* L.  Boraginaceae  ALCG 173  Comfrey  Quaker Comfrey, boneset | herb  cultivated in  garden | introduced  Europe, West Asia ^9^ | 0.12/ 0.12 | 0.06/ 0.13 | leaves (B) | | rheumatism (C)  headache (C)  prostate complaints (Z) | | infusion/ oral (C)  concoction with Rosmarinus officinalis in decoction/ oral (Z) |
| *Cannabis sativa* L.  Cannabaceae  ALCG 172  Marihuana  Marijuana,Hemp,Cannabis | herb  cultivated | introduced  North West of Himalayan mountains, Asia^7^ | 0.12/ 0.00 | 0.19/ 0.06 | leaves (B) | | rheumatism (B) | | concoction with Justicia filibracteolata in extract in alcohol^U2^/ oral (B),  concoction with Aristolochia ringens, Erythroxylum coca and Justicia filibracteolata in extract in alcohol/ topical and oral (B)  maceration in extract in alcohol/ topical and oral (Z)^U2^ |
| *Clusia aff. ellipticifolia* Cuatr.  Clusiaceae or Guttiferae  ALCG 122  Gaque | small tree  cultivated  in an edge of  pastures plot | native  Colombian Andes and Amazons^5^ | 0.08/ 0.00 | 0.13/ 0.00 | fruits (C)  leaves (Z) | | against warts (C)  back pain (Z) | | fresh fruit juice/ topical directly on skin (C)  heated leaves/ topical directly on the back (Z) |
| *Tradescantia zebrina* Heynh.  Commelinaceae  ALCG 115  Suelda con suelda roja or cinta  Wandering jew | herb  cultivated in  small garden | introduced  Neotropical Guatemala and Mexico^13^ probably Panamá^8^ | 0.04/ 0.08 | 0.00/ 0.00 | leaves and flowers (Z) | | diabetes (Z)  rheumatism (Z) | | decoction/ oral (Z)  maceration/ topical-emplast (Z) |
| *Callisia monandra* (Sw.) Schult. & Schult f.  Commelinaceae  ALCG 118  Suelda con suelda blanca  Cojite morado | herb  wild in edge of secondary forest with enought shadow and moisture | native to Caribean territories^20^ including Colombia | 0.12/ 0.00 | 0.19/ 0.13 | leaves and stems (B) | | fracture bones (B)  rheumatism (Z) | | maceration/ topical-emplast (B)  maceration/ topical-emplast (Z)  decoction/ topical-baths (Z) |
| *Sechium edule* (Jacq.) Sw.  Cucurbitaceae  ALCG 151  Guatilla  Alligator pear,pear squash | climbing vine  plant  cultivated in  homegarden | introduced  Central America and Mexico ^8^ | 0.08/ 0.08 | 0.06/ 0.00 | fruit (B) | | hipertension complaints (B) | | decoction/ oral (B) |
| *Cupresus lusitanica* Mill.  Cupressaceae  ALCG 129  Pino ciprés  Mexican cypress,Cedar of Goa | tree  cultivated in  small garden | introduced Europe^7^  naturalized in Mexico^24^ | 0.28/ 0.04 | 0.13/ 0.00 | branches (B) | | rheumatism (B)  caughs and  asthma (B)  against bledder complaints in  children (C)  tiredness and weakness (Z)  kidney complaints (Z) | | decoction/ topical-baths (B)  concoction with Eucalyptus globulus and Solanum nigrum in concentrated extract by decoction/ oral (B)  maceration/ topical-emplast (C)  concoction with Eucalyptus globulus in decoction/ topical-baths (Z)  decoction/ oral (Z) |
| *Equisetum giganteum* L.*  Equisetaceae  ALCG 156  Cola de caballo  horsetail | herb  wild in  strem edge | native  Central and South America Central part of Colombian Andeans^7^ | 0.28/ 0.00 | 0.19/ 0.00 | leaves and stem (B)*2  root (C) | | skin infections and inflamations (B)  kidney complaints (B)  againts rheumatism  and osteoporosis (C) | | decoction/ topical-baths (B)  decoction/ oral (Z)  decoction/ oral (B)  concoction with Furcraea macrophylla in decoction/ baths (C) |
| *Erythroxylum coca* Lam.  Erythroxylaceae  ALCG 73  Coca | Shrub or  small tree  Cultivated  in solar | native  Colombia and  South  America^5^ | 0.08/ 0.08 | 0.44/ 0.25 | leaves and branches (B)  leaves (C)  leaves (B)  leaves (Z) | | rheumatism (B)  rheumatism (C)  tiredness and weakness (B)  relaxant (B)  tooth pain (Z) | | concoction with Cannabis sativa, Aristolochia ringens and Justicia filibracteolata in extract in alcohol/ topical and oral (B)  decoction/ oral (C)  infusion/ oral (B)  fresh maceration/ topical on the tooth (Z) |
| *Croton Funckianus* Muell. Arg.  Euphorbiaceae  ALCG 120  Sangregado | tree  wild in edge of route | native  Colombian Andes^5^ | 0.00/ 0.00 | 0.06/ 0.06 | leaves (C) | | tooth pain (C) | | maceration/ topical-emplast (B) |
| *Mimosa pudica* L.  Fabaceae  ALCG 175  Dormidera  Sensitive Plant, Mimosa | herb  wild in open  pastures | native  South and Central America^7^ | 0.12/ 0.12 | 0.56/ 0.00 | leaves (B) | | tooth pain (B)  rheumatism (C)  for helping children to fall asleep (C) | | fresh maceration/ topical-emplast (B)  decoction/ topical-bath (C) |
| *Senna obtusifolia* (L.) Irwin & Barnaby  Fabaceae  ALCG 161  Alcaparro  Sicklepod | shrub  spontaneous  in a sideroad | introduced  48 lower states, Virgin Islands,Puerto Rico^23^ | 0.12/ 0.08 | 0.50/ 0.06 | branches (B)  leaves (C)  branches (C)  branches (B) | | fever (B)  gripe (C)  headache (C)  for healing uterus after giving birth (C)  to stop noise bleeding in children (Z) | | decoction/ topical-bath (C)  concoction with Trichanthera gigantea and Cestrum mariquitense in decoction/ topical-bath (Z)  decoction/ oral (C)  fresh extract by maceration/ topical-massage or bath (C)  decoction/ topical-bath (C)  decoction in milk/ topical-bath (Z) |
| *Juglans neotropica* Diels  (e-CH/Z)  Juglandaceae  ALCG 67  Cedro Nogal  Colombian walnut | tree  cultivated in solar  within a secondary forests well drened | native  Amazons, Orinoquia  Central Andes Range^7^ | 0.40/ 0.20 | 0.00/ 0.00 | leaves (Z)  leaves and branches (Z)  leaves (Z) | | allergies (Z)  diaper rash and other  skin complaints  as acne and feets' fungus (Z)  uterus cleaning (Z) | | infusion/ oral (Z)  decoction/ topical in baths (Z)  decoction/ topical in hip baths (Z) |
| *Melissa officinalis* L.* º  Lamiaceae  ALCG 92  Toronjil  Common balm | herb  cultivated in  homegarden | introduced  Europe and Asia^3^ | 0.88/ 0.72 | 0.75/ 0.62 | leavesº2 and  stems (B)*2 | | gripe (B)  fevers (B)  relaxant (B)  stomach ache (B)  intestine complaints (B) | | decoction/ oral (B) or fresh extract by maceration in water/ oral (B) or infusion/ oral (B) |
| *Ocimum campechianum* Mill.  Lamiaceae  ALCG 74  Albahaca  Basil, Wild mosquito plant | herb  cultivated in  homegarden | native  North, Central and Northern South  America^23^ | 0.28/ 0.16 | 0.63/ 0.31 | leaves (B)  seeds (B)  leaves (Z) | | Intestinal complaints (B)  eye dust (B)  gripe (Z) | | infusion/ oral (B)  one seed is placed in the eye/ topical (B)  infusion/ oral (Z) |
| *Mentha viridis* L.  Lamiaceae  ALCG 91  Menta  Spearmint | herb  cultivated in  homegarden | introduced  Europe ^3^ | 0.36/ 0.20 | 0.50/ 0.38 | leaves (B) | | gripe (B)  stomach ache (B)  inflammations of  intestine (B) | | infusion/ oral (B)  infusion or decoction/ oral (B)  fresh extract by maceration in water/ oral (C)  infusion/ oral (B) |
| *Satureja brownei* (Sw.) Briq.  Lamiaceae  ALCG 130  Poleo | herb  cultivated in  small garden | native  South America, Colombian Andean  Ranges^3^ | 0.60/ 0.20 | 0.19/ 0.06 | leaves and stems (B) | | against bledder complaints in children (B)  condiment herb (B)  stomach ache (Z)  enhance blood cleaning (Z) | | maceration/ topical-emplast (B)  combination with food/ oral (B)  infusion/ oral (Z)  infusion/ oral (Z) |
| *Origanum majorana* L.*  Lamiaceae  ALCG 76  Mejorana  knotted marjoram,sweet marjoram | herb  cultivated in  solar | introduced  Asia menor^13^ | 0.36/ 0.28 | 0.06/ 0.00 | leaves (Z)*2 | | stomach aches and intestine complaints (Z)  prostata complaints (Z)  kidney complaints (Z) | | infusion or decoction/ oral (Z)  infusion/ oral (Z)  infusion/ oral (Z) |
| *Rosmarinus officinalis* L.* º  (e-Z)  Lamiaceae  ALCG 149  Romero  Rosemary | woody herb  cultivated in  homegarden | introduced  Península Ibérica, Western Mediterranean^3^ | 0.44/ 0.44 | 0.31/ 0.19 | leaves (B)  * 2 º 2  leaves and stems (B) | | lungs complaints (B)  tooth pain (B)  hipertension complaints (B)  against hair loss (B)  muscles pain (C) rheumatism (C)  stomach ache (Z)  tiredness (Z) | | infusion/ oral (C)  decoction in milk/ oral (Z)  maceration/ topical-emplast on tooth (B)  infusion/ oral (B)  fresh extract by maceration/ topical-massage (C)  decoction/ topical-baths (Z)  fresh extract by maceration/ topical-massage (C)  infusion/ oral (Z) |
| *Mentha suaveolens* Ehrh.  Lamiaceae  ALCG 127  Yerbabuena blanca  Round leaved mint | herb  cultivated in  homegarden | introduced  Europe^3^ | 1.20/ 1.04 | 0.94/ 0.94 | leaves (B)  shoots (Z)  leaves and stems (Z)  leaves (Z)  leaves and stems (Z) | | stomach ache (B)  headaches (B)  intestinal inflamations (B)  intestinal parasits (Z)  against infections and inflamations (Z)  fevers (Z)  menstrual cramps (Z)  diaper rash (Z) | | infusion or decoction/ oral (B)  fresh extract by maceration/ oral (B)  infusion or fresh extract by maceration/ oral (B)  concoction with Allium sativum  cloves, in concentrated extract by decoction/ oral (Z)  concoction with Piper cf.bogotense,  Trichanthera gigantea and Phytolacca rivinoidesin decoction/ topical-baths (Z)  infusion/ oral (Z)  concoction with Ambrosia cumanensis and Ruta graveolens in decoction/ oral (Z)  decoction/ topical-baths (Z) |
| *Persea americana* Mill.  Lauraceae  ALCG 100  Aguacate  Avocado, butter pear | tree  cultivated in solar | native  Central and South  America^7^ | 0.04/ 0.04 | 0.25/ 0.13 | shoots (C)  shoots (Z)  fruit (C)  fruit (Z) | | gripe (C)  apendicitis (C)  sensitive gums (Z)  against dry hair (C)  regulator of digestion (Z) | | decoction and concoction with fresh extract of Verbena littoralis and Citrus limon juice/ oral (C)  decoction/ oral (C)  maceration/ topical (Z)  maceration/ topical-massage (C)  combination with food/ oral (Z) |
| *Allium fistulosum* L.  Liliaceae  Cebolla larga  Scallion, green onion | gregarious  herb  cultivated in  homegarden | introduced  Siberia ; Altai montains in Siberia^7^ | 0.12/ 0.08 | 0.31/ 0.19 | roots (B)  leaves (B)  leaves (B)  young leaf (C)  roots (C)  leaves (B) | | intestinal obstipation (B)  stomach ache (B)  condiment herb (B)  against intestinal obstipation in newborns (C)  lost of memory (C)  fever (Z) | | decoction/ oral (B)  fresh extract by maceration/ oral (C)  decoction/ oral (Z)  combination with food (B)  fresh leaf as suppository/ rectal (C)  decoction/ oral (C)  maceration/ topical-emplast on head-front (Z) |
| *Allium sativum* L. * º  Liliaceae  Ajo  Garlic | herb  cultivated in  pod | introduced  Southwestern Asia; Central Asia ^7^ | 0.32/ 0.04 | 0.12/ 0.06 | bulbus (B)  *2 º 2 | | gripe (C)  fevers (C)  againts intestinal worms (C)  againts amebiasis (Z)  rheumatism (Z) | | concoction with alcohol-Aguardiente U2/ oral (C)  decoction/ oral (C)  concoction with alcohol-Aguardiente-U2/ oral (Z)  concoction with shoots of Menta suaveolens in concentrated extract by decoction/ oral (Z)  concoction with Justicia filibracteolata and Aristolochia ringens in extract in alcohol ^U1^- (Z) |
| *Althaea officinalis* L. º  Malvaceae  Malva  Marsh mallow | herb  cultivated  in pod | introduced  Mediterranean^5^ | 0.08/ 0.04 | 0.38/ 0.00 | leaves (C)  flowers (C)  leaves (B) | | kidney complaints (C)  caugh (C)  stomach  ache (Z)  fever (Z) | | infusion/ oral (C)  fresh extract by maceration/ oral (Z)  infusion/ oral (Z) |
| *Anoda cristata* (L.) Schl.  Malvaceae  ALCG 170  Patechula (Campo Hermoso)  Escobo (Zetaquira)  Spuded anoda,crested anoda | herb  wild in open  pastures | introduced  Mexico and part of Central America^5^ | 0.00/ 0.00 | 0.19/ 0.13 | leaves and stems (C) | | fever (C)  healing wounds and againts spots (C) | | concoction with Senna obtusifolia in extract from maceration/ topical-bath or rub body (C)  fresh extract by maceration/ topical-rub skin (C)  decoction/ topical-bath (C) |
| *Hibiscus rosa-sinensis* L.  Malvaceae  ALCG 164  Rosado  Hibiscus | shrub and  groundcover  cultivated in  small garden | introduced  China^11,13^ | 0.12/ 0.08 | 0.31/ 0.19 | leaves (C)  leaves and flowers (C)  leaves (Z)  flowers (Z) | | intestinal obstipation (C)  fever (C)  againts hair loss (C)  nervous system tonic (Z)  eye inflamations (Z) | | fresh extract by maceration/ oral (C)  concoction with Cestrum mariquitense in decoction/ topical-bath (C)  decoction/ topical-bath (C)  fresh extract by blending/ oral (Z)  fresh extract in water/ topical-eye-bath (Z) |
| *Psidium guineense* Sw.  Myrtaceae  ALCG 121  Guayabo Zapatero or Cimarrón  Brazilian guava, wild guava | small tree  spontaneous  in a private  pastures plot | native  Tropical America, native to Colombia^5^ | 0.12/ 0.08 | 0.44/ 0.31 | leaves and branches (B) | | skin infections and  recover wounds (B)  intestinal complaints (B)  against umbilical hernia (C)  rheumatism (C)  against colds and cramps in uterus (C) | | decoction/ topical-baths (C)  maceration/ topical-emplast (Z)  decoction or infusion/ oral (B)  infusion/ oral and decoction/ topical-baths (C)  decoction/ topical-baths (C)  concoction with other eight bitter plants in decoction/ topical-steam baths (C) |
| *Eucalyptus cf. globulus* Labill. * º  Myrtaceae  ALCG 128  Eucalipto  Gum tree, Silver-leaved  mountain gum | tall tree  wild in edge of  secondary forest | introduced  South East of Australia and Tasmania ^6 ,10^ | 0.56/ 0.00 | 0.44/ 0.19 | leaves (B)* 2  º 2  branches (C)  leaves (B)  branches (B)  leaves (Z) | | gripe (B)  fevers (C)  caughs and asthma (B)  rheumatism (B)  tiredness and weakness (Z) | | infusion/ oral (B)  concoction with Jacaranda copaia and Allium sativum in decoction/ topical-baths (C)  concoction with fresh maceration-Jaccaranda copaia and Allium sativum in cloves extract in alcohol^U2^ / oral (C)  concoction with Solanum nigrum and Cupresus lusitanica in concentrated extract by decoction/ oral (Z)  decoction/ topical-baths (B)  concoction with Cupresus lusitanica in decoction/ topical-baths (Z) |
| *Cattleya schroederae* Rchb. F.  Orchidaceae  ALCG 165  Lirio  Easter orchid, Baron schroeder´s  cattleya | Epiphytic herb  cultivated in  solar | native  Colombia^8^ | 0.00/ 0.00 | 0.69/ 0.63 | leaves (C) | | typhus (C)  denge (C)  fever (C)  intestinal  complaints (C) | | fresh extract by maceration of one leafe/ oral (C)  extract by blending in concoction with water/ oral (C)  fresh extract by maceration of a piece of leafe in concoction with water/ oral (C) |
| *Petiveria alliacea* L. *  Phytolaccaceae  ALCG 136  Anamú  Anamú plant | herb  cultivated in small garden | native  Andean(specially Peruvian Amazonas), Caribean and South east of Colombia ^7^ | 0.00/ 0.00 | 0.25/ 0.25 | leaves (C)*2 | | stomach cancer (C)  against lacerations in mouth (C)  headache (C) | | infusion/ oral (C)  decoction/ oral (C)  extract by maceration/ topical-rinsing mouth out/ (C)  infusion/ oral (C) |
| *Phytolacca rivinoides* Kunth & C.D.Bouché  Phytolaccaceae  ALCG 146  Guaba, Cargamanto | shrub  spontaneous  in a solar | native  Central and South America, Colombian Andeans^7^ | 0.08/ 0.00 | 0.38/ 0.25 | leaves and branches (B) | | rheumatism (C)  erisipela (C)  inflamations and skin infections (C)  inflamations and skin infections (Z) | | decoction/ topical-baths (C)  concoction with Mentha suaveolens,Piper bogotense and Trichanthera giganteain decoction/ topical-baths (Z) |
| *Piper hispidum* Sw.  Piperaceae  ALCG 85  Cordoncillo negro  Jamaican pepper | shrub  cultivated in  solar/it can also occur in wild | native  Central and South America and native to Caribean territories^20^ | 0.00/ 0.00 | 0.44/ 0.38 | leaves and stems (C) | | high blood presure (C)  fevers (C)  tiredness and weakness (C)  rheumatism (C) | | fresh extract by maceration in water/ oral (C)  concoction with Verbena littoralis in decoction/ topical-bath (C)  decoction / topical-bath (C),steam baths/ topical(C) |
| *Piper cf. bogotense* C.DC.  Piperaceae  ALCG 81  Cordoncillo blanco | shrub  cultivated in  solar/it can also occur in wild | native  North of South America ^12^ | 0.08/ 0.00 | 0.50/ 0.25 | leaves (C)  leaves and  stems (Z) | | high blood presure (C)  fevers (C)  mouth dryness (C)  skin infections and inflamations (Z) | | fresh extract by maceration in water/ oral (C)  extract in water/ oral (C)  concoction with Mentha suaveolens and Phytolacca rivinoides in decoction/ topical-bath (Z) |
| *Plantago major* L.*  Plantaginaceae  ALCG 374  Llantén  Common plantain | herb  spontaneous  in homegarden | introduced  Europe and Asia^3^ | 0.32/ 0.16 | 0.81/ 0.31 | leaves (B)*2 | | eye dust and infections (B)  gastritis (B)  liver cleanser (B)  kidney complaints (C)  fever (C)  eczema (C)  healing wounds (Z) | | decoction/ topical eye bath (C)  fresh extract by destilation in glass bottle/ topical-eye drops (Z)  fresh extract by maceration/ oral (B)  decoction/ oral (C)  fresh extract by maceration/ oral (Z)  decoction/ oral (C)  decoction/ oral (C)  fresh maceration in concoction with honey/ topical-emplast (C)  concoction with Calendula officinalis and Solanum nigrum in decoction/ topical-baths (Z) |
| *Cymbopogon citratus* (DC.) Stapf.*  Poaceae  ALCG 77  Limonaria or limoncillo  West Indian,Lemon grass,  oil grass | herb  cultivated in  solar | introduced  India ² | 0.56/ 0.60 | 0.44/ 0.50 | leaves (B)*2 | | stomach aches (B)  intestine complaints (Z)  gripe (Z)  prostata complaints (C)  fevers (C) | | infusion or decoction/ oral (B)  infusion/ oral (Z)  decoction/ oral (Z)  decoction/ oral (C) |
| *Rumex crispus* L.  Polygonaceae  ALCG 148  Romaza, Lengua de vaca  Curled dock | herb  wild in open  pastures | introduced  Europe^7^, West Asia ^20^ | 0.08/ 0.04 | 0.19/ 0.00 | leaves (B) | | fevers (B)  kidney complaints (C)  liver cleanser (C)  intestinal inflamations (Z) | | fresh extract by maceration/ oral (C)  fresh extract by maceration/ topical-baths (Z)  fresh extract by maceration/ oral (C)  heated leaves/ topical directly on the lower belly (Z) |
| *Rubus glaucus* Benth.  Rosaceae  ALCG 126  Moras  Andean blackberry | sub-shrub  cultivated in  homegarden | native from Mexico to Ecuador and from center to south of Colombian Andeans^7^ | 0.28/ 0.00 | 0.50/ 0.13 | shoots and fruits (B)  shoots (Z) | | rheumatism (B)  against skin infections and inflamations (C)  against rashes (C)  against spots (C)  caughs (Z) | | decoction/ oral and decoction/ topical-baths (B)  decoction/ topical-baths and  infusion/ oral (C)  decoction/ topical-baths (C)  decoction/ oral (Z) |
| *Citrus aurantium* var. Amara L.  Rutaceae  Naranjo agrio  Bitter orange,Seville orange | small tree  cultivated in  homegarden | introduced  Southern Vietnam^13^ , Southeast Asia^5^ | 0.20/ 0.20 | 0.69/ 0.25 | fruit (B)  leaves (Z) | | relaxant (B)  headache (C)  mouth infections (B)  rheumatism (B)  body dryness (Z) | | steam baths/ topical (B)  infusion/ oral (C)  decoction/ topical-for rinsing mouth (B)  steam baths/ topical (C)  decoction/ topical-baths (Z)  decoction/ oral (Z) |
| *Citrus maxima* (Burm. ex Rumph.) Merr.  Rutaceae  ALCG 98  Limón mandarin  Pommelo, Pumelo | small tree  cultivated in  homegarden | introduced  South East  Asia ^5,13^ | 0.36/ 0.24 | 0.25/ 0.19 | fruits (B)  shoots (C)  fruits (B)  fruits and branches (C)  fruits (Z)  fruits and branches (Z) | | gripe (B)  stomach ache (B)  inflamations because infections (C)  headache (Z)  fevers (Z)  rheumatism (Z) | | juice,alone or in combination with aguadepanela-U3-/ oral (B)  decoction of shoots/ oral (C)  juice in combination with water / oral (B)  decoction / topical-baths (C)  juice in combination with water/ oral (Z)  decoction/ topical-baths (Z) |
| *Ruta graveolens* L.*  Rutaceae  ALCG 86  Ruda | herb  cultivated in  small garden | introduced  Canarian Islands Europe meridional^5^ | 1.24/ 1.08 | 0.75/ 0.50 | leaves (B)  leaves,  flowers and stems (B)*2  leaves and stems (C)  leaves (Z) | | to strengthen uterus (B)  against cramps in uterus and menstural pains (B)  rheumatism (Z)  intestinal parasits (C)  cardioregulator (Z) | | fresh choped leaves combined  with boiled egg/ oral (B)  decoction/ oral (B)  decoction/ topical-bath (Z)  fresh extract by maceration in water/ oral (C)  infusion/ oral (Z) |
| *Citrus limon* (L.) Burm. F.  (e-Z)  Rutaceae  ALCG 93  Limón ácido  Lemon,Citrus | small tree  cultivated in  homegarden | introduced  Himalaya and Indochina^5^ | 0.52/ 0.20 | 1.00/ 0.63 | fruits (B) | | gripe (B)  fevers (B)  against infections in throat (B)  stomach complaints (B)  intestine complaints (B)  rheumatism (C)  againts excema (C)  headache (C)  to prevent high blood presure (Z) | | juice alone or in combination with water or aguadepanela/ oral (B)  juice in combination with water/ oral (B)  juice/ to gargle-topical (B)  juice in combination with water/ oral (B)  a fresh hitted-hot fruit/ to massage-topical (C)  fruit pieces mixed with salt in water/ to do baths-topical (C)  fresh fruit pulp/ to rub the head-topical (C)  juice in combination with water/ oral (Z) |
| *Physalis peruviana* L.  Solanaceae  ALCG 163  Uchuba,Guchuba  Cape gooseberry | shrub  cultivated in  homegarden | introduced  Northern South America-Peru ^3^ | 0.16/ 0.04 | 0.19/ 0.00 | fruits (B) | | eye dust and infections (B) | | fresh extract from maceration/ topical-eye drops (B)  a variation is a concoction with honey (C) |
| *Solanum nigrum* L. *  Solanaceae  ALCG 157  Yerbamora(Zetaquira)  Almoraduz(Campo Hermoso)  black night shade | sub shrub  spontaneous  in solar | introduced  Eurasia ^13,23^ | 0.20/ 0.04 | 0.19/ 0.00 | fruits and leaves (B)*2 | | diabetes (C)  inflamations and skin infections (Z)  respiratory complaints (Z) | | infusion/ oral (C)  decoction/ topical-baths (Z)  concentrated extract by decoction/ oral (Z) |
| *Cestrum mariquitense* Kunth  Solanaceae  ALCG 102  Tinto | shrub  cultivated in  solar | native  Tropical America ^13^ Boyacá and Cundinamarca-Colombia ^3^ | 0.08/ 0.08 | 0.50/ 0.25 | leaves (B) | | sinucitis (B)  fevers (B)  headache (C) | | fresh extract by maceration/ topical-noise drops (B)  fresh extract by maceration/ oral (B)  concoction with Hibiscus rosa sinenesis in decoction/ topical-baths (C)  fresh extract by maceration/ oral (C |
| *Cecropia* sp.  Urticaceae •  Cecropiaceae ^++^  Yarumo  Pumpwood, trumpet tree | tree  wild in  secondary  forest | native  Colombian Andes^7^ | 0.08/ 0.00 | 0.06/ 0.00 | leaves (Z)*2 | | cardiac complaints as hypertrophy (B) | | concoction with alcohol-dye/ oral (B) |
| *Urera baccifera* (L.) Gaudich ex Wedd.  Urticaceae  ALCG 99  Ortiga mayor, Pringamoza | herb  spontaneous in a farm with  a large patch of secondary forest | native  Central and South America Central Andeans and Orinoquia in Colombia ^7^ | 0.20/ 0.08 | 0.25/ 0.06 | leaves and branches (B)  leaves (C) | | rheumatism (B)  allergies (B)  blood circulation tonic (C) | | decoction/ topical baths (B)  infusion/ oral (C) |
| *Urtica dioica* L.* º  Urticaceae  ALCG 66  Ortiga Blanca or Ortiga menor  Great Nettle or Stinging Nettle | herb  spontaneous in a farm with  a large patch of secondary forest | introduced  eurasian, meridional-boreal, native of Britain ^18^ | 0.44/ 0.32 | 1.12/ 0.63 | leaves and stems (B)  leaves (B)*2  leaves and stems (C)  leaves (C) | | rheumatism (B)  allergies (B)  blood cleaner and tonic (B)  antihemoragic (B)  skin infection and inflamations (C)  gripe (C)  mouth dryness (C) | | fresh-maceration/ topical in cataplasm (B)  decoction / topical in baths (B)  Infusion/ oral (B)  fresh extract by maceration/ topical to rub skin (C)  infusion/ oral (C)  concoction with Jacaranda copaia and Rosmarinus officinalis in decotion/ oral (C) |
| *Lippia alba* (Mill.) N.E.Brown*  Verbenaceae  ALCG 72  Pronto alivio  Bushy,Matgrass | shrub  cultivated in  homegarden | native to Colombia ^5,25^ | 0.68/ 0.56 | 0.75/ 0.56 | leaves (B)*2 | | stomach complaints (B),  diarrhoea (B)  tiredness and weakness (C), (Z)  childbirth (Z) | | decoction / oral (B)  decoction/oral (C),infusion/oral (Z)  infusion/oral (Z) |
| *Lippia citriodora* (Lam.) Kunth*  Verbenaceae  ALCG 70  Cidrón  Lemon Scented Verbena | shrub  cultivated in  homegarden | native  Colombian Andean  range ^8^ | 0.48/ 0.36 | 0.25/ 0.13 | leaves (B)*2 | | stomach and intestinal complaints (B)  relaxant (B)  general  indisposicion (Z) | | infusion/ oral (B)  decoction/ oral (B)  infusion/ oral (Z) |
| *Lantana camara* L.  Verbenaceae  ALCG 117  Florota  Cambara de Espinto or Pricky Lantana | herb  wild in open  pastures | native to Caribean territories including Colombia, Continental US and Hawai^20^ | 0.20/ 0.08 | 0.25/ 0.25 | leaves and stems (B)  flowers (C)  flowers, stems and leaves (C)  leaves and stems (Z) | | high bilirubin in newborns (B)  hepatitis (C)  yellow fever (C)  hepatitis (Z) | | decoction/ topical-baths (B)  infusion/ oral (C)  decoction in milk/ oral and  decoction/ topical-baths (C)  decoction/ topical-baths (Z) |
| *Verbena littoralis* Kunth *  Verbenaceae  ALCG 79  Verbena blanca  Vervain, owi | herb cultivated in  solar | native  western and central Andean ranges^5^ | 0.96/ 0.40 | 0.88/ 0.63 | leaves and  stems (B) | | fevers (B)  gripe (B)  rheumatism (Z)  rheumatism (C)  tiredness and weakness (C) | | decoction/ topical baths (B)  fresh extract by maceration in water/ oral (Z)  concoction with Calendula officinalis and Piper hispidum in decoction/ topical baths (C)  concoction with Piper hispidum in decoction/ topical in baths (C)  infusion/ oral (Z) |
| *Viola odorata* L.*  Violaceae  ALCG 111  Violeta  Sweet violet/common violet | Acaule herb  Cultivated in  small garden | introduced  Europe, Austral Asia and Boreal Africa ^5^ | 0.04/ 0.00 | 0.00/ 0.00 | leaves*2 and stems (Z) | | cough (Z)  asthma (Z) | | infusion/oral (Z) |
| | B: Both municipalities; C: Campo Hermoso; Z: Zetaquira | | |  | |  | |  |  |  |  |  | | --- | --- | --- | --- | --- | --- | --- | --- | --- | --- | --- | --- | | ^U1^ A glass bottle is filled with plant(s) parts, red grape wine usually and, it is buried under the ground during a month | | | | | | | | |  |  |  | | ^U2^ A similar process as it is described in U1, with the variation of the use of aguardiente, which is the introduced alcoholic drink similar to spirit (snaps) | | | | | | | | | | |  | | ^U3^ Aguadepanela is a traditional colombian homemade drink.The ingredients are water and panela, that is an unrefined food product of which the main component is sugarcane juice. | | | | | | | | | | | | | * Reported in Colombian Vademecum, 2008;* ^2^ Plant material of interest reported in Colombian Vademecum, 2008 | | | | | | | |  |  |  |  | | º Reported in WHO,2009; º ^2^ Plant material of interest reported in WHO, 2009 | | | | | | |  |  |  |  |  | | • www.tropicos.org | | | | | | | |  |  |  |  | | ++ www.ipni.org/ipni/plantnamesearchpage.do | | | | |  | |  |  |  |  |  | | (e-CH/Z) Endangered plant species according to the perceptions of the locals in Campo Hermoso (CH) and/or Zetaquira (Z) | |  | |  |  | |  |  |  |  |  | | | ¹ Rothmaler Werner, 1994 | | --- | | ² Antolinez González J. C. et.al ,2008 | | ³ Garcia Barriga Tomo III1992 | | ⁴ De Fraume Melida, 1988 | | ⁵ Garcia Barriga Tomo II,1992 | | ⁶ Fonnegra,2007 | | ^7^ Garcia Barriga Tomo I 1992 | | ^8^ Perez-Arbelaez 1978 | | ^9^ Centro de Educacion No Formal FUNIBA | | | ^10^ Carlos Paez Perez, 1964 | | --- | | ^11^ www.Botanical.com | | ^12^ www.zipcodezoo.com/plants/ | | ^13^ www.en.wikipedia.o | | ^14^ www.mansfeld.ipk-gatersleben.deg | | ^15^ www.flowersofindia.net | | ^16^www.mobot.org | | ^17^ Soloiki M et al, 2008 | | ^18^ www.botanicus.org (Missouri Botanical Garden) | | | | | | | ^19^ www.issg.org (global invasive species database) | | --- | | ^20^www.cbif.gc.ca/itis (Integr. Taxonomi Inf. Sys.) | | ^21^ Missouri Bot. Garden 2007 in Colomb. Vademecum | | ^22^  a.o. (tropicalforages.info) | | ^23^ http://plants.usda.gov/java/profile?symbol=SEOB4# | | ^24^ http://www.conifers.org/cu/cup/lusitanica.htm | | ^25^ Vera et. al. in Revista Cubana de plantas medicinales 2010 | | ^26^ http://zipcodezoo.com/Key/Plantae/Cecropia_Genus.asp | | ^27^ http://plants.jstor.org/flora/flos003208 | | | | |  |  | |  |  | | | | | | | | | | |
